# Non-invasive characterization of pericyte dysfunction in mouse brain using functional ultrasound localization microscopy

**DOI:** 10.1038/s41551-025-01465-x

**Published:** 2025-07-30

**Authors:** Jérémy H. Thalgott, Nicolas Zucker, Thomas Deffieux, Marit S. Koopman, Alexandre Dizeux, Cristina M. Avramut, Roman I. Koning, Hans-Jurgen Mager, Ton J. Rabelink, Mickaël Tanter, Franck Lebrin

**Affiliations:** 1 Einthoven Laboratory for Vascular and Regenerative Medicine, Department of Internal Medicine (Nephrology), Leiden University Medical Centre, Leiden, the Netherlands; 2 Institute Physics for Medicine Paris, INSERM U1273, ESPCI Paris-PSL, CNRS UMR8063, Paris, France; 3 Department of Cell and Chemical Biology, Electron Microscopy Facility, Leiden University Medical Centre, Leiden, the Netherlands; 4 Department of Pulmonology St Antonius Hospital, Nieuwegein, the Netherlands

**Keywords:** Cerebrovascular disorders, Ultrasonography

## Abstract

Early microscopic-scale pericyte dysfunction contributes to the initial stages of many neurological diseases and represents a strong candidate target for therapeutic intervention. A non-invasive imaging modality able to image microvascular alterations induced by pericyte dysfunction is needed. In addition, the development of pericyte-focused therapies remains challenging due to the lack of early biomarkers of disease progression. Here we show that cerebral microvascular alterations induced by pericyte dysfunction can be characterized non-invasively in mice using functional ultrasound localization microscopy (fULM). Depletion of endothelial endoglin in adult mice as a model of hereditary haemorrhagic telangiectasia, leads to pericyte detachment in the arteriole–capillary transition (ACT) zone. Imaging reveals that arteriolar capillaries have irregular shapes, increased diameters, reduced blood speed and neurovascular uncoupling mainly localized in the ACT zone. Transforming growth factor-β signalling activator C381 restores pericyte coverage and neurovascular response. Our study underscores the potential of fULM in characterizing early microvascular alterations. As super-resolution ultrasound transitions to the clinic, our data support its future use in monitoring pericyte-focused therapies in humans.

## Abstract (web summary)

An optimized pipeline for functional ultrasound localization microscopy is safely and non-invasively applied to detect and characterize early microvascular alterations with high spatial and temporal resolutions in mouse.

## Main

Central nervous system (CNS) pericytes are mural cells located within the basement membrane of small-sized blood vessels, positioned between endothelial cells and perivascular astrocytic endfeet^1^. Their interactions with the endothelial cells are crucial for the development of healthy blood vessels. Pericytes regulate angiogenesis^2^, the formation and maintenance of the blood–brain barrier (BBB)^3,4^, the entry of immune cells to the brain parenchyma^3^ and cerebral blood flow (CBF)^5–13^.

**Fig. 1:**
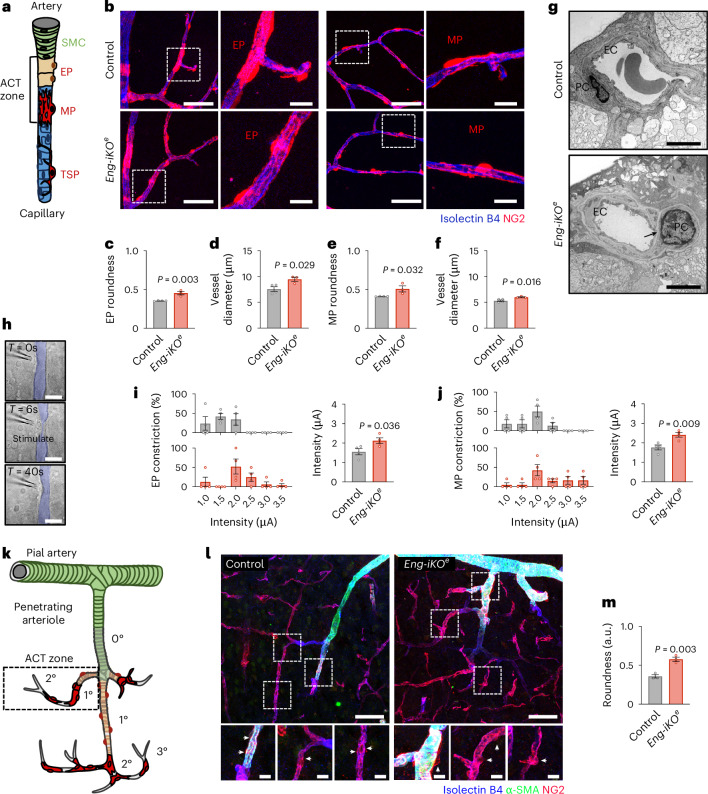
Mural cell organization in *Eng-iKO*^*e*^ mouse retinal and cerebral vasculature. **a**, Schematic of mural cell populations along the arterial–capillary axis. **b**, Confocal images of genetically labelled retinal EPs and MPs (NG2DsRedBAC transgenic mice, red) attached to endothelium (isolectin-B4, blue) in control and *Eng-iKO*^*e*^ mice. Scale bar, 50 μm. Higher magnification of EPs and MPs in boxes shown at right. Scale bar, 10 μm. **c**, Quantification of EP roundness and **d**, capillary diameters in control (*n* = 3, total pericytes = 67) vs *Eng-iKO*^*e*^ (*n* = 3, total pericytes = 46) mice. **e**, Quantification of MP roundness and **f**, capillary diameters in control (*n* = 3, total pericytes = 95) vs *Eng-iKO*^*e*^ (*n* = 3, total pericytes = 72) mice. **g**, TEM image of a retinal vessel with a contractile pericyte in *Eng-iKO*^*e*^ mice. Scale bar, 2 μm. The black arrow indicates the basal membrane space located between an endothelial cell (EC) and a pericyte (PC). **h**, Vascular segment with a contractile pericyte before, during and after electrical stimulation. Scale bar, 20 μm. **i**,**j**, Left: proportion of EPs (**i**) and MPs (**j**) inducing vascular constriction at the indicated current (μA). Right: the mean current inducing EP- and MP-mediated contraction in control (*n* = 4, total EPs = 14, MPs = 16) and *Eng-iKO*^*e*^ (*n* = 4, total EPs = 15, MPs = 19) mice. **k**, Schematic of mural cells enwrapping penetrating arterioles across the cortex. **l**, Representative confocal images of the post-arteriole transition zone of penetrating arterioles in control and *Eng-iKO*^*e*^ brains (isolectin-B4 for endothelial cells, blue; NG2 for pericytes, red; α-SMA for SMCs and EPs, green). Scale bar, 50 μm. Higher magnification of contractile pericytes in boxes shown at the bottom. White arrows mark contractile pericytes Scale bar, 10 μm. **m**, Quantification of pericyte roundness in control (*n* = 3, total pericytes = 70) and *Eng-iKO*^*e*^ (*n* = 3, total pericytes = 53) mice. Error bars show s.e.m. *P* values result from unpaired two-tailed Student’s *t*-tests. Source data

There are four distinct types of mural cell along the arteriovenous axis (Fig. 1a). Smooth muscle cells (SMCs) cover arterioles^14^, while three distinct types of pericyte enwrap blood capillaries (Fig. 1a). Ensheathing pericytes (EPs) which exhibit a hybrid morphology resembling both SMCs and pericytes are found at the arteriole–capillary transition (ACT) zone. Capillaries are covered by pericytes that display mesh (MP) and thin-strand processes (TSP). All types of pericyte are contractile and therefore can modulate blood flow, but they operate on different timescales and respond to different stimuli. EPs at the ACT zone are very dynamic and can rapidly induce blood vessel dilatation in response to changes in brain activity. This provides rapid and local increases in blood perfusion (functional hyperaemia) in regions of higher neuronal activity^5–11^. The ACT zone dilates earlier and slightly faster than the neighbouring arterioles, revealing that the EPs are first to respond during neurovascular coupling^7,9,12^. Capillary pericytes are necessary to maintain basal capillary tone. Their contractile properties also suggest a role in slow modulation of capillary diameter in response to changes in brain activity^13^, although their contribution during neurovascular coupling remains unclear.

Early pericyte dysfunctions commonly occur with aging^15,16^ and are associated with the development of various neurological diseases including Alzheimer’s disease^17^, stroke^12^, migraine^18^, epilepsy^19^, diabetes^20^, radiation necrosis^21^ or Huntington’s disease^22^. Pericyte alterations include early constriction of capillaries and pericyte death or migration away from the endothelium. These pathological responses can lead to long-lasting hypoperfusion, loss of BBB function and increase of immune cell entry to the brain which together can contribute to neuronal damage.

Albeit rare, blood vessels of patients with hereditary haemorrhagic telangiectasia (HHT) exhibit characteristics seen in other cerebrovascular diseases such as dilated thin-walled vascular structures prone to bleeding^23–25^. Brain-related clinical symptoms include migraine, seizures, strokes, abscesses, intracranial haemorrhages and the presence of focal neurologic deficits^26^. HHT affects 1 in 5,000 people worldwide and is caused by mutations in the *ENG* (endoglin, HHT1) and *ACVRL1* (activin-like receptor kinase 1, HHT2) genes. They encode receptors of transforming growth factor-β (TGFβ) and bone morphogenetic protein (BMP) family ligand, which are expressed in endothelial cells. Despite knowing the causative gene mutations for decades, there is still limited understanding of how the gene mutations impact the different vascular segments along the arteriovenous axis and the haemodynamic consequences for the brain. Interestingly, thalidomide was shown to reduce blood vessel weakness caused by HHT by enhancing pericyte recruitment to the endothelium in both mice and humans^21,23^, suggesting that pericyte-focused therapies may benefit other cerebrovascular diseases. However, for this therapeutic approach to be effective in conditions not defined by genetic markers such as HHT, early detection of disease progression can be crucial.

Obtaining functional information on living organs non-invasively across different size scales is a tremendous challenge in clinical imaging, as pericyte dysfunction starts locally and deep into the brain before expressing large-scale and observable symptoms. Structural and functional changes of the microvasculature are often discrete and include increased blood capillary dilatation and tortuosity, variations in vessel density and changes in local blood flow and permeability^4,15^. Clinical imaging modalities such as computed tomography (CT), magnetic resonance angiography (MRA) or functional magnetic resonance imaging (fMRI) are essential for the evaluation of patients with cerebrovascular pathologies; however, they are restricted to tenths of millimetres in spatial resolution and thus fail to detect the vast majority of cerebral small vessels and quantify most blood flow dynamics. Recent advancements in functional ultrasound localization microscopy (fULM) with the ability to detect brain vasculature and function at microscopic resolution have contributed substantially to angiography^27^. However, despite its great promise, fULM has had several important limitations, including its use on anaesthetized rats with trepanation, which restraints its potential clinical translation.

Here we have optimized the entire experimental pipeline, including injection methods, ultrasonic sequences and post processing to demonstrate that fULM can be safely and non-invasively applied to detect and characterize early microvascular alterations with high temporal (2 s) and spatial resolutions (6.5 µm) (Supplementary Fig. 1) in a mouse model of HHT, in which endothelial endoglin is deleted in adult mice following tamoxifen injection (*Eng-iKO*^*e*^) (Supplementary Fig. 2a). First, we investigated mural cell function along the arteriovenous axis by combining optical imaging, transmission electron microscopy (TEM) and functional electrophysiology methods. We also quantified the architectural and haemodynamic changes at rest and during sensory stimulation using non-invasive fULM. Furthermore, we explored therapeutic options for treating HHT including C381, an agonist of the TGFβ signalling pathway that has shown promise in treating neurological diseases^28^, and VAD044, an allosteric AKT inhibitor currently in trials for HHT. Our findings demonstrate the potential of non-invasive fULM to uncover insights into pericyte-induced cerebral small-vessel disease, particularly those linked to vascular alterations caused by HHT and to evaluate drug efficacy and mechanism of action.

## Results

### *Eng* gene deletion alters pericyte function of the ACT zone

We administrated tamoxifen to 3-month-old *Cdh5-cre*^*ERT2*^;*Eng*^*fl/fl*^(*Eng-iKO*^*e*^);*NG2DsRedBAC* mice which have mural cells labelled in red and examined the retinal and brain vasculature at day 4 after gene deletion (Supplementary Fig. 2a). Loss of endothelial endoglin protein was confirmed by immunofluorescence staining (Supplementary Fig. 2b,c). Confocal images showed that the retinal blood vessels appeared unchanged in *Eng-iKO*^*e*^ mice, resembling that of control mice with the three interconnected capillary layers remaining intact (Supplementary Fig. 3a). No differences in vessel area, vessel length or branch points could be observed between control and *Eng-iKO*^*e*^ mice (Supplementary Fig. 3b,c). The superficial plexus was composed of six major arteries and veins (Supplementary Fig. 4a) that branched into arterioles and venules that were connected to a capillary network in direct contact with the intermediate and deep layers (Supplementary Fig. 3a). The arterial network showed regular coverage by α-smooth muscle cells (α-SMCs) that were circumferentially aligned, and the expression of α-smooth muscle actin (α-SMA) remained intact in *Eng-iKO*^*e*^ mice (Supplementary Fig. 4a). TEM failed to reveal any apparent abnormalities in the morphology of the SMCs and confirmed the intact coat of the distal arterioles (Supplementary Fig. 4b). By contrast, EPs and MPs of the ACT zone in *Eng-iKO*^*e*^ mice showed a more rounded soma with a limited number of circumferential processes enwrapping capillaries (0.35 ± 0.004 a.u. versus 0.45 ± 0.04 a.u. for EPs and 0.41 ± 0.003 a.u. versus 0.48 ± 0.07 a.u. for MPs in control compared to *Eng-iKO*^*e*^ mice) (Fig. 1b,c,e), despite both genotypes having a similar number of pericytes (Supplementary Fig. 3d). Blood capillaries were also significantly enlarged at the EP or MP soma midline (7.57 ± 0.89 µm versus 9.47 ± 0.70 µm and 5.32 ± 0.30 µm versus 6.01 ± 0.16 µm, respectively), confirming the partial detachment of EPs or MPs of the ACT zone (Fig. 1d,f), while the vascular density and the number of branch points were found to be identical between the genotypes (Supplementary Fig. 3b,c). TEM analysis confirmed that pericytes in the superficial layer of *Eng-iKO*^*e*^ mice were poorly associated with the endothelium, with an enlarged basement membrane with space between the two cell types in comparison with control mice (Fig. 1g and Supplementary Fig. 5). To assess whether the deletion of *Eng* in endothelial cells alters the ability of mural cells to regulate capillary diameter, we stimulated retinal SMCs and pericytes electrically by applying a pipette on their soma to induce a vascular constriction^29^. Electrical stimulation applied to all studied SMCs induced arteriolar constriction in both control and *Eng-iKO*^*e*^ mice, with current intensities ranging from 1 to 2.5 µA and mean intensities of 1.84 ± 0.29 µA and 1.90 ± 0.29 µA, respectively (Supplementary Fig. 4c). Electrical stimulation applied to EPs and MPs also induced capillary constriction. The required current intensities used to stimulate EPs were slightly lower than that used to stimulate MP responses, ranging from 1 to 2 µA and from 1 to 2.5 µA, respectively (Fig. 1h–j). It correlated with high and low to undetectable α-SMA content (Supplementary Fig. 3e). In *Eng-iKO*^*e*^ mice, most EPs and MPs constricted capillaries only when high current intensities were applied to the soma, and some pericytes even did not respond at all. Mean intensities were 2.13 ± 0.29 µA for EPs and 2.41 ± 0.23 µA for MPs in *Eng-iKO*^*e*^ mice, while they were 1.55 ± 0.31 µA for EPs and 1.76 ± 0.26 µA for MPs in control mice (Fig. 1h–j). TSPs of the capillaries did not exhibit any morphological differences, the number of pericytes was unchanged, and capillaries had normal diameters between the two genotypes (Supplementary Fig. 6a–d). TSPs were insensitive to electrical stimuli (Supplementary Fig. 6e).

TEM images confirmed the tight association through direct contacts between endothelial cells and TSPs in both control and *Eng-iKO*^*e*^ mice (Supplementary Fig. 6f). MPs and TSPs in the capillaries are known to have vital roles for BBB maintenance and small-molecule transport^4^. To assess this, we injected Cadaverine Alexa Fluor-555 into the circulation and analysed potential extravasation into retinal parenchyma. EP and MP detachment in *Eng-iKO*^*e*^ mice did not induce altered vessel permeability (Supplementary Fig. 6g). We next examined the contractile functions of mural cells in *Eng-iKO*^*e*^ mice 5 weeks after gene deletion. Our findings revealed that both EP and MP phenotypes worsened, and all studied SMCs now required higher current intensities to induce vessel constriction (Supplementary Fig. 7a–d).

The general features of the retinal vasculature are structurally and functionally similar to those of the cerebral vasculature, although it exists as a more extended arteriole to venule network in the retinal circulation^30^. In the cortex, pial arteries branch into penetrating arterioles, both of which are covered by SMCs that extend to arteriolar segments (ACT zones) covered by EPs and MPs marking the beginning of the capillary network (Fig. 1k). We confirmed that pericytes in the ACT zones of penetrating arterioles were much more rounded in the cortex of *Eng-iKO*^*e*^ mice than in control mice (0.36 ± 0.04 a.u. versus 0.58 ± 0.05 a.u.) (Fig. 1l,m).

These results establish that contractile pericytes in the ACT zone exhibit a significant sensitivity to the *Endoglin* gene deletion; furthermore, they indicate that mural cell detachment in both the arterial and ACT zones progresses over time potentially leading to blood flow dysregulation.

### fULM reveals rapid flow velocity changes upon *Eng* deletion

**Fig. 2:**
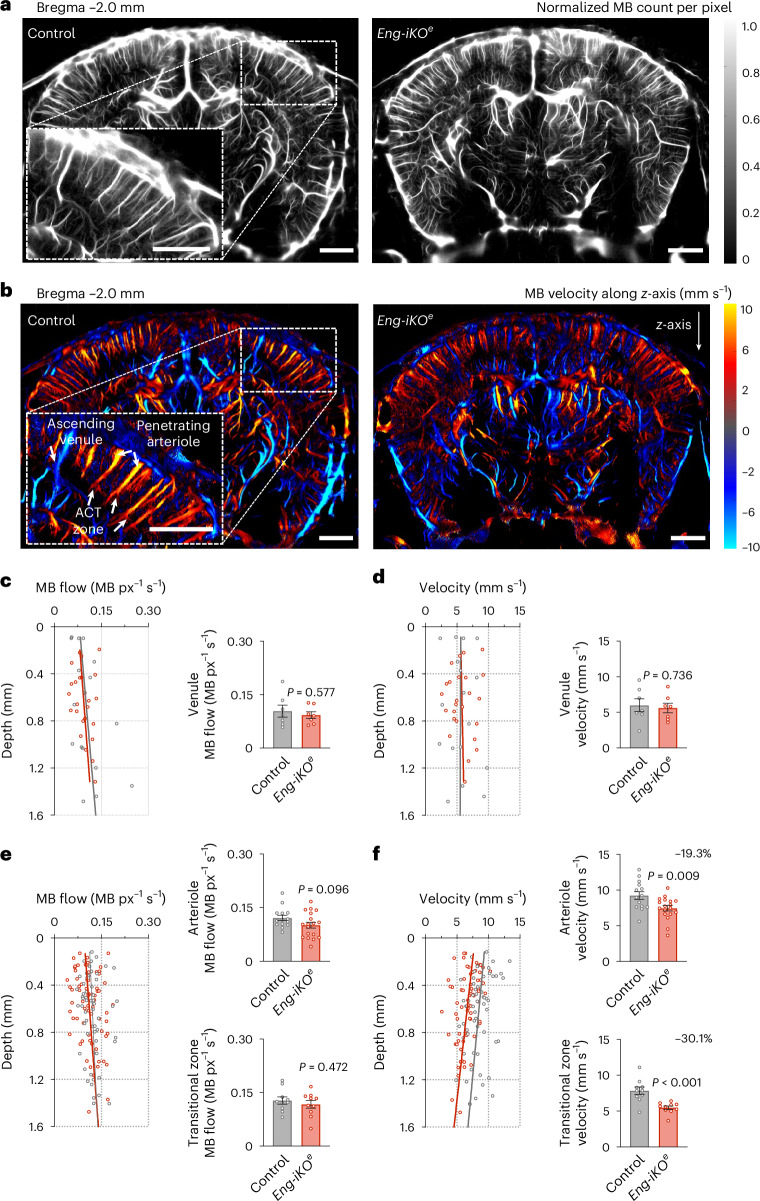
ULM reveals cerebral arteriole abnormalities in *Eng-iKO*^*e*^ mice. **a**, Normalized MB count of ULM maps of the mouse brain vasculature in both control and *Eng-iKO*^*e*^ mice. Scale bars, 1 mm. **b**, Blood flow velocity of ULM maps in both control and *Eng-iKO*^*e*^ mice. A higher magnification image in the box shows post-capillary small venules branching to an ascending venule and penetrating arterioles branching to post-arteriole transitional zone capillaries. Scale bars, 1 mm. **c**,**d**, Longitudinal profiles (left) and quantification (right) of MB count (**c**) and velocity (**d**) along ascending venules (depth 0–1,400 μm of the cortex) during the resting period in a control mouse (*n*_venule_ = 7) and in an *Eng-iKO*^*e*^ mouse (*n*_venule_ = 7). **e**,**f**, Longitudinal profiles of MB count (**e**) and velocity (**f**) along penetrating arterioles (left) and quantification of MB flow and velocity along arterioles (depth 0–400 μm of the cortex) (top right) and post-arteriole transitional blood vessels (depth 800–1,400 μm of the cortex) (bottom right) during the resting period in both a control mouse (*n*_arteriole_ = 14 and *n*_ACT_ = 10) and an *Eng-iKO*^*e*^ mouse (*n*_arteriole_ = 18 and *n*_ACT_ = 10). Error bars represent s.e.m. *P* values result from unpaired two-tailed Student’s *t*-tests. Source data

The capillary bed structure in the cerebral cortex area exhibits remarkable conservation between mice and humans^31^. Both species feature a consistent branching pattern connecting the feeding arterioles to the draining veins. There are 3 to 4 diverging bifurcations wrapped with EPs and MPs, collectively referred to as the ACT zone, followed by 3 to 4 converging bifurcations that connect to the draining venules. To assess haemodynamic parameters of the various vascular compartments with a brain-wide field of view at a microscopic resolution, we injected a contrast agent under the form of microbubbles (MBs) into the blood circulation of control and *Eng-iKO*^*e*^ mice at day 4 after gene deletion. The flow of these MBs in the cerebral vasculature was evaluated transcranially using a high-frequency ultrasonic probe. Ultrafast imaging using 11 compounded plane wave transmissions at a 5,500 Hz pulse repetition frequency (PRF) enabled continuous ultrasound image acquisition at a high frame rate of 500 Hz. Singular value decomposition of consecutive blocks of 200 ultrasound images separated MB signatures from the tissue^32^. Localizing MBs in each image, tracking their trajectories both in space and time, and accumulating millions of them enabled the extraction of local density and velocity vector field images with a spatial resolution of 6.5 µm and a temporal resolution of 2 s (Supplementary Fig. 1). During data acquisition, whiskers were intermittently brushed in short periods for 25 repetitions of 30-s whisker stimulations. These stimuli led to an increase in the number of MBs detected in this area, indicating enhanced local perfusion in response to neuronal activation (hyperaemia)^27^. We accumulated the positions and speeds of the MBs over the patterns and used temporal sliding windows on the previous set of images to construct dynamic maps of MB flow. This approach allowed us to differentiate MBs during resting and stimulation periods within the same acquisition, enabling the study of both basal haemodynamic and brain-wide responses to whisker stimuli using the same animals (Supplementary Fig. 8a). Baseline maps of MB flow were computed by counting MBs detected in each pixel during the rest periods, while velocity maps were computed as the mean velocity of these bubbles in each pixel during the rest periods (Supplementary Fig. 8b). Two-dimensional (2D) maps of total MB count (Fig. 2a) and average speed (Fig. 2b) revealed most of the cerebral vascular organization with vessel diameters ranging from 70 µm down to 10 µm in both a control and an *Eng-iKO*^*e*^ mouse. Arterioles and venules from the upper right cortex were discriminated by analysing the sign of the speed in the vertical *z*-axis (Supplementary Video 1).

We were able to decipher pial arteries and descending arterioles, down to second to third-order blood capillaries and venules to ascending veins. We did not observe any major architectural vascular distinctions between the two genotypes in this representative dataset (Fig. 2a,b). For each blood vessel, manual measures of the speed at various depths were then extracted from a profile line orthogonal to the vessel (Supplementary Fig. 8c,d). Quantitative analyses of the MB flow and velocities suggested that endoglin depletion did not alter the haemodynamic of the venous system when measured across the entire cortical depth in the *Eng-iKO*^*e*^ mouse (Fig. 2c,d). By contrast, MB velocity appeared to be decreased in the arterioles and ACT zones of the *Eng-iKO*^*e*^ mouse (−19.3% and −30.1% compared with the control mouse, respectively) based on fULM measurements, while MB flow remained unchanged, indicating a dilation of the descending arterioles and pre-capillary arterioles (Fig. 2e,f). Interestingly, the phenotype of the descending arterioles was more pronounced in the deep layers of the cortex (depth range of 0.4–1.6 mm) corresponding to ACT zones where blood vessels are covered by EPs and MPs (Fig. 1k,l). Owing to the large field of view of our imaging modality, simultaneous analyses could be obtained for every descending arteriole and ascending vein and capillary across the whole-mouse brain slice image. On average in the right cortex of each mouse, we analysed 15 descending arteries, 5 ascending veins and 25 1st/2nd-order blood capillaries, providing an exemplary representation of the functional alterations of the arteriole and ACT blood vessels of the *Eng-iKO*^*e*^ mouse.

### Arterioles appear irregular in shape, influencing fluid flux

**Fig. 3:**
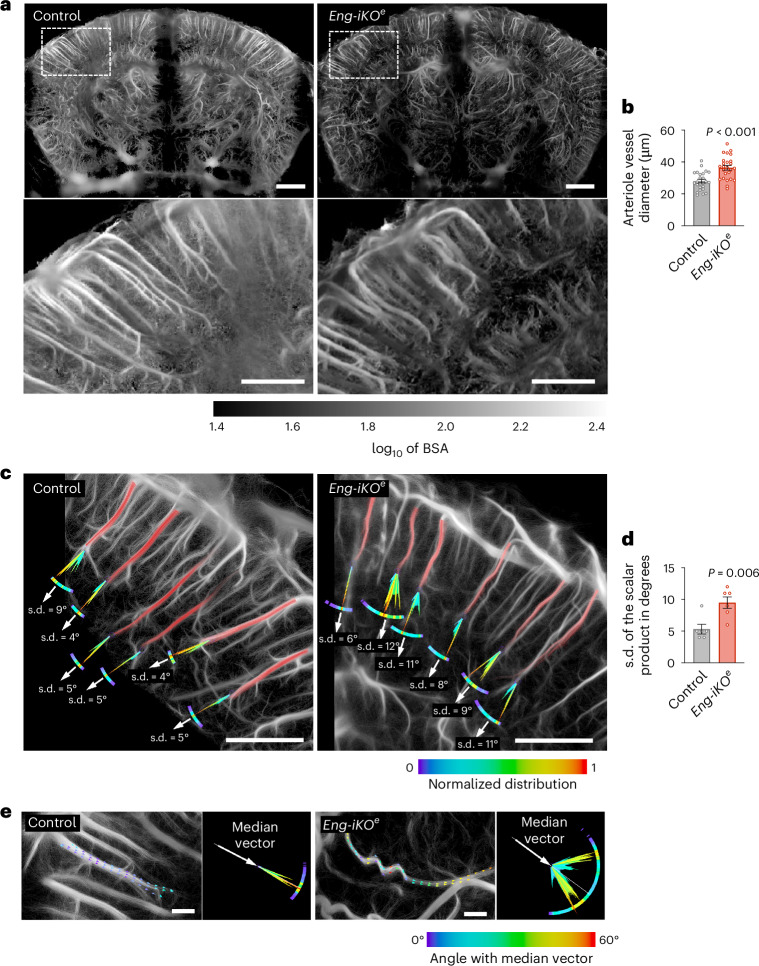
Analysis of the arteriolar architecture. **a**, ULM backscattering imaging offering better blood vessel delineation than MB count. Scale bars, 500 μm. Higher magnification images of the cortex in boxes are shown at the bottom. Scale bar, 500 μm. **b**, Quantification of arteriolar blood vessel diameter in a control mouse (*n*_vessel_ = 25) and in an *Eng-iKO*^*e*^mouse (*n*_vessel_ = 25). **c**, High magnification images of MB count maps showing vessel tortuosity. **d**, Comparison of vessels tortuosity in a control mouse (*n*_arteriole_ = 6) and in an *Eng-iKO*^*e*^ mouse (*n*_arteriole_ = 6) measured as the standard deviation of velocity vector scalar products along each vessel. **e**, High magnification images of MB count maps showing a helical buckling vascular structure in Eng-iKO^e^ mice. Scale bars, 100 μm. Error bars represent s.e.m. *P* values result from unpaired two-tailed Student’s *t*-tests. Source data

To identify possible structural anomalies, we extracted the additional backscattering information available in 2D fULM imaging^33^ (Fig. 3a). The amplitude of the backscattered echoes of each MB was registered and its mean value was computed in every pixel of the image. This approach allowed for better delineation of small-sized blood vessels compared with conventional fULM imaging with a 3D rendering effect due to the intrinsic ability of the backscattering amplitude to convey physical information about the out-of-plane distance^33^. As expected in this representative dataset, descending arterioles and the 1st to 3rd branches in the *Eng-iKO*^*e*^ mouse exhibited larger diameters compared with the blood vessels of the control mouse (36.46 ± 7.25 µm versus 28.02 ± 5.71 µm) at day 4 after gene deletion (Fig. 3b). Furthermore, arterioles in the *Eng-iKO*^*e*^ mouse exhibited deformations, including twist and helical buckling vascular structures, in contrast to the normal straight cylindrical shape observed in the control mouse, indicating loss of stability under mechanical stress (Fig. 3a). To quantify this phenotype, the tortuosity of the descending arterioles was estimated using the variation in the velocity vector field’s orientation in vessels^34^. This was particularly useful for identifying blood vessels with discrete irregular shape and tortuosity. In this example, the descending arterioles showed significantly higher metrics of tortuosity in the *Eng-iKO*^*e*^ mouse in comparison with the control mouse (Fig. 3c,d). Moreover, helical buckling vascular structures were detected in deep brain regions of the *Eng-iKO*^*e*^ mouse, but never in the control mouse (Fig. 3e and Supplementary Video 2). This outcome was expected, as pericyte detachment represents a significant destabilizing factor. Pericytes typically serve as stabilizers, regulating vascular tone and maintaining vessel integrity. In addition, pericyte-independent factors may also contribute to vessel wall weakening in HHT mice. Alterations in the composition of the extracellular matrix (ECM), particularly involving elastin and collagen have been reported in *Eng*^*+/−*^ mice^35^. Collagen provides tensile strength, while elastin imparts elasticity to the vessels. The combination of pericyte detachment and defects in the ECM probably makes arterioles more vulnerable to deformation and tortuosity when subjected to mechanical stress.

### fUS imaging reveals neurovascular coupling alterations

**Fig. 4:**
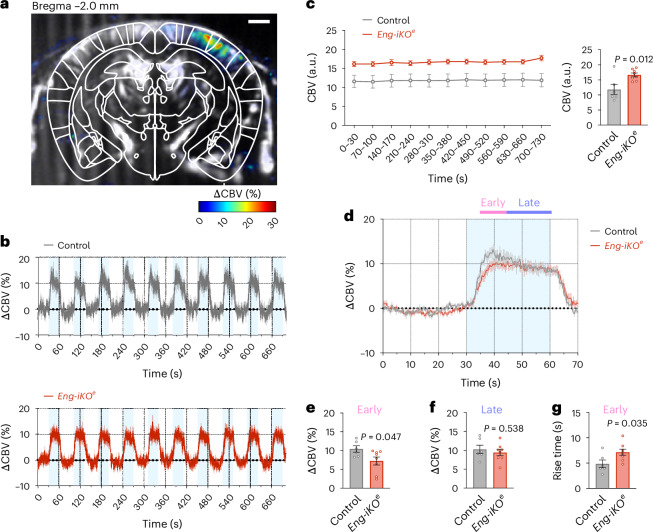
Neurovascular uncoupling revealed by fUS. **a**, Transcranial fUS imaging of task-induced changes in cerebral blood flow in the somatosensorial cortex during whisker stimulation in anaesthetized mice. Scale bar, 1 mm. **b**, Mean time-series of all trials of the activated region, with blue boxes showing temporal correlation between the stimulus pattern and the haemodynamic response in both control (*n* = 6) and *Eng-iKO*^*e*^ (*n* = 8) mice. **c**, Baseline CBV values over time and quantification in both control (*n* = 6) and *Eng-iKO*^*e*^(*n* = 8) mice. **d**, Average of the haemodynamic response within a chosen region of interest. **e**–**g**, Quantification of the increased response amplitude in both control (*n* = 6) and *Eng-iKO*^*e*^ (*n* = 8) mice at early (**e**, start to 10 s) and late (**f**, from 10 to 30 s) excitation response. **g**, Quantification of the rise time. All error bars represent s.e.m. *P* values result from unpaired two-tailed Student’s *t*-tests. Source data

To assess how EP and MP dysfunctions in the ACT zone impact the haemodynamic response to neuronal activity, we first used functional ultrafast ultrasound (fUS) imaging in control and *Eng-iKO*^*e*^ mice at day 4 after gene deletion^36,37^. We applied a series of 10 mechanical whisker stimulations, each lasting for 30 s, followed by a 40-s rest period. Each stimulation reliably generated a fUS power Doppler signal detectable at the level of single voxels in both control and *Eng-iKO*^*e*^ mice, indicative of an increase in cerebral blood volume (CBV) in the barrel cortex (Fig. 4a,b). As expected, the baseline CBV measured before each stimulation was slightly higher in *Eng-iKO*^*e*^ mice (16.76 ± 1.79 a.u.) compared with control mice (11.83 ± 4.16 a.u.). However, successive stimulations did not affect baseline levels, ruling out the possibility that changes in baseline CBV over time may influence the haemodynamic response to sensory stimulation (Fig. 4c). In control mice, an initial overshoot was observed during stimulation before the signal settled to a new steady-state value and until the stimulation end. We therefore separated the haemodynamic response into two phases: an early phase corresponding to the overshoot and a late phase representing the steady-state response (Fig. 4d). Conversely, in *Eng-iKO*^*e*^ mice, no overshoot was detected (Fig. 4d), and the initial response amplitude was lower compared with control mice (7.27 ± 3.01% versus 10.44 ± 2.06%) (Fig. 4e). By contrast, the steady-state amplitude response was similar in both control and *Eng-iKO*^*e*^ mice (Fig. 4f). In addition, the time to reach the maximal amplitude response was delayed compared with control mice (7.15 ± 1.73 s versus 4.87 ± 1.85 s) (Fig. 4g). We also quantified the haemodynamic response at day 1 after gene deletion when there was no evidence of pericyte dysfunction (Supplementary Fig. 9a). As anticipated, no differences in the amplitude response or in the rise time were observed between both genotypes (Supplementary Fig. 9b–d), confirming that EPs and MPs of the ACT zone play a regulatory function in the initiation of the haemodynamic response to neuronal activation.

### fULM detects ACT zone dysfunctions during stimulation

**Fig. 5:**
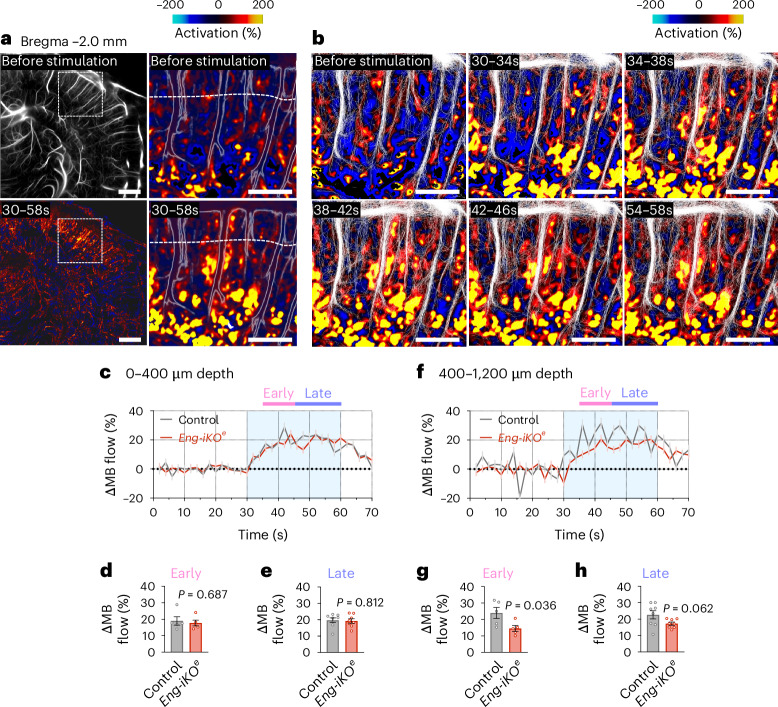
Contractile pericyte dysfunction during the hyperaemic response revealed by fULM. **a**, Top left, MB count ULM map at bregma = −2 mm containing the barrel cortex. Bottom left, corresponding fULM activation map showing the activated region in the barrel cortex. Scale bar, 1 mm. Top right, zoom-in of the fULM activation map showing the absence of hyperaemia before stimulation in the 24–28 s time window. Scale bar, 500 μm. Dashed white line corresponds to *z* = 400 μm depth in the cortex. Bottom right, zoom-in of the fULM activation map during the stimulation (30–58 s). Scale bar, 500 μm. **b**, Activation maps and MB tracks detected in 4-s temporal windows during stimulation. Scale bar, 500 μm. **c**, Mean time-series of MB flow increase from 0 to 400 μm depth. **d**,**e**, Quantification of the averaged MB flow increase (control *n* = 4, *Eng-iKO*^*e*^ *n* = 6) mice from 0 to 400 µm depth in early (*n* = 5 samples) and late (*n* = 8 samples) phase of the stimulation. **f**, Mean time-series of MB flow increase from 400 to 1,200 μm depth. **g**,**h**, Quantification of the averaged MB flow increase (control *n* = 4, *Eng-iKO*^*e*^ *n* = 6) mice from 400 to 1,200 µm depth in early (*n* = 5 samples) and late (*n* = 8 samples) phase of the stimulation. All error bars represent s.e.m. *P* values result from unpaired two-tailed Student’s *t*-tests. Source data

To gain insights into the spatial and temporal dynamics of functional hyperaemia in *Eng-iKO*^*e*^ mice, MBs detected during fULM acquisition were averaged within each spatial voxel (6.5 µm × 6.5 µm) and time window (2 s) and the results were then averaged over the 25 stimulation patterns. This process is analogous to block design processing in fMRI and resulted in the creation of a dynamic fULM movie that tracks the spatiotemporal hyperaemia during an averaged whisker stimulation pattern at microscopic scale (Fig. 5a and Supplementary Fig. 10a–d). During the early phase of stimulation (30–34 s), fULM revealed a significant activation (indicated by a relative increase in MB count) that was spatially localized around the arterioles, confirming the pivotal role of the ACT zones in the initiation of the hyperaemic response (Fig. 5a). This localized initial hyperaemia later extended from deep cortical regions to the cortical surface during sustained whisker stimulation (30–60 s) (Fig. 5a). While this initial response amplitude was observed in the ACT areas deeply situated within the cortex and in the parenchyma surrounding the descending arterioles (corresponding to regions controlled by pericytes), there was relatively less activation detected within the arteriolar compartment driven by SMC. The representation of raw individual MB tracks detected in successive 4-s temporal windows during stimulation demonstrated an increase in MB detection events throughout the stimulation phases (Fig. 5b). Neighbouring arteriolar compartments in the barrel cortex were not all activated (Fig. 5a), emphasizing a plausible spatial optimization of the vascular network response^38^. The wide field of view offered by fULM compared with two-photon imaging enabled the examination of diverse responses in multiple distinct vascular compartments within a single experiment. The MB flow activation profile for arteriolar segments covered by SMC, located from the cortical surface and down to 400 µm deep did not exhibit any significant difference between control and *Eng-iKO*^*e*^ mice (Fig. 5c–e). Conversely, the same activation profile for the region located below depth *z* = 400 µm that correspond to the ACT zones showed a lower initial response amplitude associated with a longer time to reach the maximal amplitude response in *Eng-iKO*^*e*^ mice compared with control mice (Fig. 5f–h). Our findings revealed the spatial localization of the altered vascular compartments and confirmed the selective dysfunction of the ACT zones using non-invasive ultrasound.

### Impaired active TGFβ bioavailability in *Eng-iKO*^*e*^ mice

**Fig. 6:**
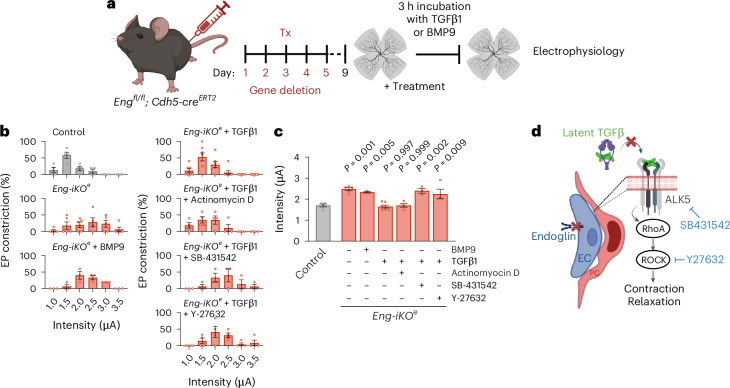
Impaired TGFβ bioavailability in *Eng-iKO*^*e*^ mice. **a**, Schematic showing adult induction of *Eng* deletion in endothelial cells and ex vivo retina treatments. Tx, tamoxifen **b**, Proportion of EPs inducing a vascular constriction at the indicated current (μA) and **c**, mean intensity inducing EP-mediated vessel constriction in the different conditions. At day 9, retinas of *Eng-iKO*^*e*^mice were collected and incubated for 3 h in medium containing TGFβ1 (5 ng ml^−1^) (*n* = 5, total EPs = 30) or vehicle alone (*n* = 5, total EPs = 26) in the absence or presence of actinomycin D (1 μg ml^−1^) (*n* = 4, total EPs = 21), SB431542 (10 μM) (*n* = 3, total EPs = 15) or Y27632 (100 μM) (*n* = 4, total EPs = 22) that inhibits the transcription, ALK5 or ROCK, respectively. Pericytes were then subjected to electrical stimulation. Retinas of control mice (*n* = 4, total EPs = 18) were in medium alone. **d**, TGFβ modulates pericyte vasocontraction/relaxation. TGFβ is produced in an inactive form by both endothelial cells and pericytes. Activation of TGFβ requires direct interactions between these two cell types. In *Eng-iKO*^*e*^ mice, pericytes establish only loose connections with the endothelium, leading to a lack of active TGFβ availability. This causes a reduced ALK5-mediated RhoA and ROCK activity within pericytes, leading to their relaxation. All error bars represent s.e.m. *P* values result from one-way ANOVA and Dunnett’s post hoc tests comparing the mean of each group to the control group. Source data

While HHT is currently recognized as a disease associated with reduced BMP9-, BMP10-, ALK1- and endoglin-mediated SMAD1/5 signalling in endothelial cells rather than a disease of the TGFβ signalling as initially thought^39^, the question whether TGFβ has a role in HHT remains unknown. We isolated retinas from *Eng-iKO*^*e*^ mice at day 4 after gene deletion and treated them ex vivo with active BMP9 or TGFβ1 for 3 h (Fig. 6a). Subsequently, EPs were electrically stimulated to induce capillary constriction (Fig. 6b,c). Pericytes of *Eng-iKO*^*e*^ mice pre-incubated with TGFβ1 but not with BMP9 constricted blood vessels at a mean intensity similar to that of control mice, thus restoring their contractile properties. Simultaneous incubation with actinomycin D, a potent inhibitor of transcription, did not counteract the effects of TGFβ1 in *Eng-iKO*^*e*^ retinas. By contrast, both SB431542 and Y27632, which inhibit ALK5 (also known as TGFβ receptor type 1) and ROCK signalling, respectively, blocked the effects of TGFβ1, causing pericytes to induce vessel constriction with a mean intensity similar to that of untreated *Eng-iKO*^*e*^ retinas (Fig. 6b,c). Our findings revealed that impaired active TGFβ bioavailability in the intercellular space between endothelial cells and pericytes in *Eng-iKO*^*e*^ mice leads to reduced ALK5-mediated ROCK signalling in pericytes. This reduction is responsible for their partial detachment and inability to maintain proper blood vessel calibres (Fig. 6d).

### fULM distinguishes VEGF-AKT and TGFβ roles in *Eng-iKO*^*e*^ mice

**Fig. 7:**
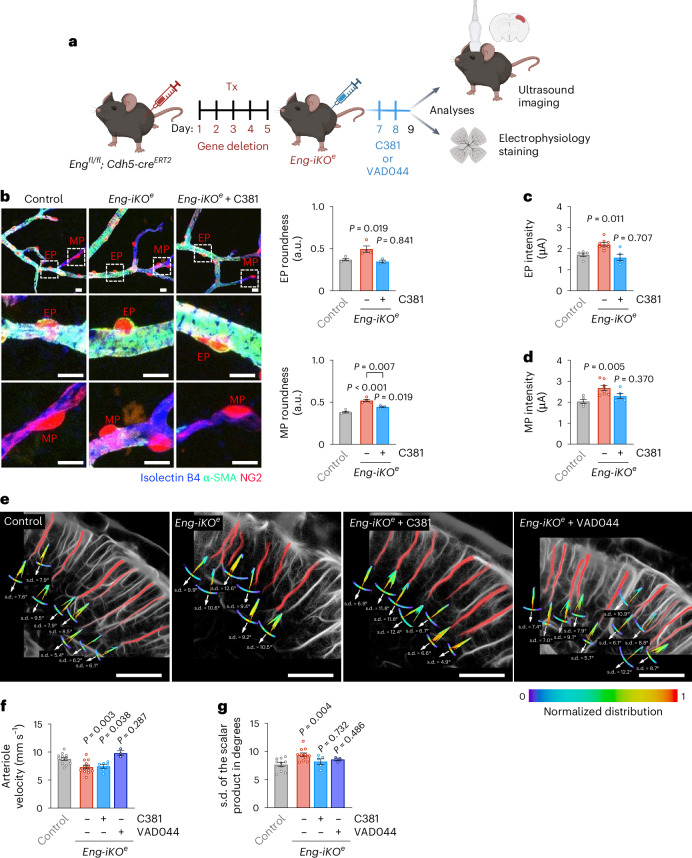
C381 drug restores the pericyte attachment velocity and tortuosity phenotypes. **a**, Diagram of the injection scheme. **b**, Left: confocal images of genetically labelled retinal EPs and MPs (*NG2DsRedBAC* transgenic mice, red) attached to endothelium (isolectin-B4, blue) in control, *Eng-iKO*^*e*^ and *Eng-iKO*^*e*^ mice treated with C381. Scale bars, 50 μm. Higher magnification of EPs and MPs in the boxes shown at the bottom Scale bars, 10 μm. Right: quantification of EP and MP roundness in control (*n* = 4, total EPs = 24, MPs = 37), *Eng-iKO*^*e*^ (*n* = 4, total EPs = 23, MPs = 57) and *Eng-iKO*^*e*^ mice treated with C381 (*n* = 3, total EPs = 29, MPs = 46). **c**, Mean intensity inducing EP-mediated contraction in control mice (*n* = 5, total EPs = 49), *Eng-iKO*^*e*^ mice (*n* = 8, total EPs = 46) and *Eng-iKO*^*e*^ mice treated with C381 (*n* = 5, total EPs = 24). **d**, Mean intensity inducing MP-mediated contraction in control (*n* = 5, total MPs = 41), *Eng-iKO*^*e*^ mice (*n* = 8, total MPs = 46) and *Eng-iKO*^*e*^ mice treated with C381 (*n* = 5, total MPs = 21). **e**, High magnification images of MB count maps showing vessel tortuosity. Scale bars, 500 μm. **f**, Quantification of MB velocity in arterioles in control (*n* = 12, total vessels = 200), *Eng-iKO*^*e*^ (*n* = 13, total vessels = 249), *Eng-iKO*^*e*^ mice treated with C381 (*n* = 6, total vessels = 202) and *Eng-iKO*^*e*^ mice treated with VAD044 (*n* = 3, total vessels = 45). **g**, Quantification of arteriolar tortuosity in control (*n* = 10, total vessels = 77), *Eng-iKO*^*e*^ (*n* = 11, total vessels = 77), *Eng-iKO*^*e*^ mice treated with C381 (*n* = 5, total vessels = 41) and *Eng-iKO*^*e*^ mice treated with VAD044 (*n* = 3, total vessels = 23). All error bars represent s.e.m. *P* values result from one-way ANOVA and Dunnett’s post hoc tests comparing the mean of each group to the control group. Source data

Restoration of TGFβ signalling would thus have therapeutic potential in HHT by improving blood vessel stability. To test this hypothesis, we injected adult *Eng-iKO*^*e*^ mice with C381 for 2 consecutive days, starting at day 2 after gene deletion (Fig. 7a). C381 has been reported to induce TGFβ signalling in the brain and importantly to exhibit neuroprotective effects in various preclinical mouse models of neurodegenerative diseases. The neuroprotective properties of C381 are not fully understood but might depend on its potent anti-inflammatory properties^28,40^. C381 treatment was sufficient to preserve EP and MP attachment to the retinal blood vessels (Fig. 7b). Electrical stimulation applied to EPs or MPs of *Eng-iKO*^*e*^ mice treated with C381 confirmed their ability to constrict blood capillaries with mean intensities similar to those applied to control mice (Fig. 7c,d). However, C381 was more effective at preserving EPs than MPs (Fig. 7b–d). Baseline maps of MB flow and velocity were generated during the resting period and the tortuosity of descending arterioles was measured as described (Fig. 3c,d). While C381 treatment successfully prevented vessel tortuosity in *Eng-iKO*^*e*^ mice, it had no significant effect on the dilatation of the descending arterioles (Fig. 7e–g). Increased VEGF–PI3K–AKTI signalling has been implicated in the development of HHT lesions^41,42^. To explore this, we tested VAD044, an AKT allosteric inhibitor currently in clinical trials for HHT. We found that VAD044 effectively prevented both blood vessel dilatation and vessel tortuosity in *Eng-iKO*^*e*^ mice (Fig. 7e–g), but it was unable to restore pericyte attachment to the endothelium (Supplementary Fig. 11).

**Fig. 8:**
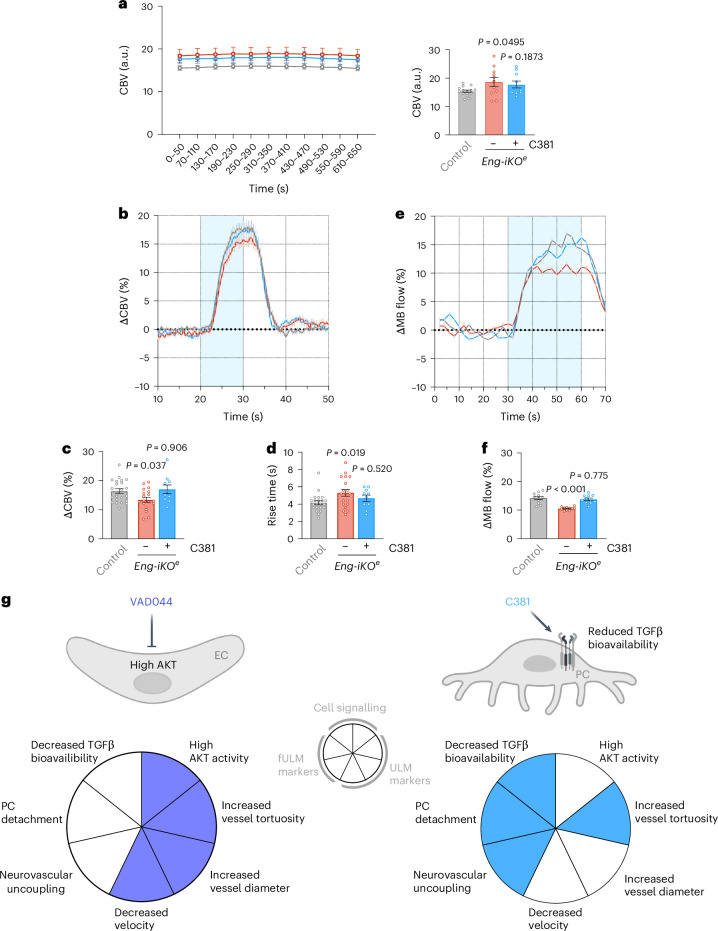
C381 drug restores haemodynamic response. **a**, Quantification of the baseline CBV in control (*n* = 15), *Eng-iKO*^*e*^ (*n* = 11) and *Eng-iKO*^*e*^ mice treated with C381 (*n* = 10). **b**, Average of the haemodynamic response within a chosen region of interest in control (*n* = 15), *Eng-iKO*^*e*^ (*n* = 11) and *Eng-iKO*^*e*^ mice treated with C381 (*n* = 10). **c**,**d**, Quantification of the increased response amplitude (**c**) and of the rise time (**d**) was performed in control (*n* = 15), *Eng-iKO*^*e*^ (*n* = 11) and *Eng-iKO*^*e*^ mice treated with C381 (*n* = 10). **e**,**f**, Mean time-series of MB flow increase (**e**) and quantification of the MB flow increase in control (*n* = 11), *Eng-iKO*^*e*^ (*n* = 11) and *Eng-iKO*^*e*^ mice treated with C381 (*n* = 11) (**f**). **g**, Mechanisms of action by which VAD044 and C381 restore blood vessel dysfunction, as revealed by fULM. All error bars represent s.e.m. *P* values result from one-way ANOVA and Dunnett’s post hoc tests comparing the mean of each group to the control group. Source data

We next investigated the haemodynamic changes induced by C381 during sensory stimulation. Although C381 treatment did not affect the baseline CBV (Fig. 8a), C381-treated *Eng-iKO*^*e*^ mice exhibited an initial response similar to control mice. The treatment restored both the time to reach the peak and the amplitude of the response as assessed using both fUS (Fig. 8b–d) and fULM (Fig. 8e,f) technologies. Our findings reveal a property of C381—its ability to restore pericyte attachment to the vessel wall by modulating TGFβ signalling in pericytes. Importantly, we demonstrate that transcranial fULM imaging effectively monitored this restored function.

## Discussion

Although studies conducted in the past few years have shed considerable light on the cellular mechanisms controlling brain haemodynamics, the lack of imaging modalities capable of non-invasively visualizing discrete and fleeting events deep into organs at the micrometre scale has limited our understanding of how microcirculation continually adapts to respond to metabolic demands in both healthy and pathological conditions. Because diseases often start locally at the cellular level before expressing large-scale and observable symptoms, the development of imaging modalities monitoring the whole brain vasculature and function down to micrometre scale resolution will not only benefit early diagnosis and treatment but will also support the development of future therapies targeting early cellular dysfunction.

Here we report that fULM can be applied non-invasively for evaluating structural and functional alterations of the microcirculation found in cerebral small-vessel diseases such as HHT and at very early stage. Our results indicate that our fULM pipeline provides a method for assessing distinct vascular phenotypes across multiple dimensions, including the different vascular compartments along the arteriovenous axis, at baseline and during functional response to neuronal activation, the vascular architecture including diameter and tortuosity, and under different therapeutic treatments. Our findings showcase the ability of non-invasive fULM to differentiate vascular phenotypes driven by distinct signalling pathways and vascular cells while effectively characterizing specific therapeutic effects (Fig. 8g). In this study, the advancement of fULM has been instrumental in elucidating the role of TGFβ in HHT, effectively resolving years of debate within the research community. It has opened avenues for the development of pericyte-focused therapies targeting TGFβ signalling.

Using HHT as a complex disease where a single gene deletion can alter multiple signalling pathways in both endothelial cells and pericytes, all of which contribute to the overall phenotype, we show that deleting the *Eng* gene in endothelial cells leads to a rapid detachment of a specific subpopulation of pericytes located in the ACT zone. Our data confirm the key role played by the ACT zone in the development of microvascular disease^43^.

At the molecular level, we found that *Eng* deletion in HHT resulted in impaired active TGFβ bioavailability within the intercellular space between endothelial cells and pericytes, which in turn affected their attachment, the arteriolar architecture and the haemodynamics during sensory stimulation. TGFβ is primarily secreted by vascular cells in a latent and inactive form, with its activation being mediated by various signals, including integrins, thrombospondin and proteases. Although the mechanisms leading to TGFβ activation in the intercellular space between endothelial cells and pericytes are not fully understood, our findings suggest that the depletion of endoglin in endothelial cells is sufficient to disrupt this process. As a result, pericytes exhibit reduced ALK5-mediated ROCK signalling, which contributes to their partial detachment and their inability to maintain proper blood vessel calibres.

Our findings underscore that HHT is a condition with defects in BMP9–BMP10–ALK1–endoglin and VEGF signalling pathways in endothelial cells^44–46^, and altered TGFβ signalling in pericytes. VAD044 treatment that targets AKT activity prevented blood vessel dilatation and tortuosity, while it was unable to enhance pericyte attachment.

By contrast, we found that treatment with C381, a potent activator of TGFβ signalling, significantly restored pericyte coverage of the microvasculature, arteriole tortuosity and the cortical neurovascular coupling response in *Eng-iKO*^*e*^ mice. C381 is a first-in-class small-molecule TGFβ activator that has shown therapeutic potential to ameliorate disease in models of neurodegeneration. While the anti-inflammatory and neuroprotective properties of C381 have already been demonstrated, notably its ability to restore lysosomal function^28^, we provide proof-of-concept data showing that C381 might also exhibit neuroprotective effects by targeting pericytes.

Recently, transcranial ULM has been demonstrated in humans for non-invasive angiography at microscopic scales^47^. This suggests that the clinical translation of fULM for monitoring cerebral vascular function during the treatment of HHT and other pericyte-related diseases may soon become a reality. To accommodate transcranial imaging in humans, the ultrasonic frequency must be decreased from 15 MHz used in rodents to 6 MHz for transfontanellar imaging in human neonates and 2 MHz for transcranial imaging in adult humans. The adult temporal bone serves as an acceptable acoustic window for transcranial imaging at this lower frequency, and microbubble backscattered echoes can be easily detected. In addition, microbubble contrast agents are routinely employed in neurosonology to image challenging patients in several European countries and the USA. The accepted injection volume for clinical contrast imaging has been shown to be sufficient for producing high-resolution images of cerebral microvasculature in the first clinical trial of ULM technology on transcranial brain imaging^47^. The ultrasonic amplitudes and acquisition durations used in this study were carefully selected to comply with safety regulations for diagnostic imaging, including the mechanical index, spatial peak temporal average intensity and thermal index. For clinical ULM, intravenous injections were performed using the bolus method; however, this should be adapted to a continuous infusion for functional ULM imaging to ensure stable microbubble concentrations during functional activations. These considerations are actively being addressed in ongoing research and development efforts aimed at the clinical translation of fULM for diagnosing and monitoring small-vessel disease. We anticipate that these efforts will pave the way for its successful implementation in routine clinical practice.

## Methods

### Mice and tissue

This study was performed in compliance with the French and Dutch government guidelines and the directive 2010/63/EU of the European Parliament. All efforts were made to minimize the number of animals used and their suffering. The Institutional Committees for Animal Welfare of Ile de France and Leiden University Medical Centre (project numbers 2014-19_2041.01 and AVD1160020174065, respectively) approved all protocols. *Cdh5(PAC)-cre*^*ERT2*^*;Eng*^*f/f*^ mice were provided by H. M. Arthur^48^. *Cdh5(PAC)-cre*^*ERT2*^*;Eng*^*f/f*^ mice were also bred with the *NG2DsRedBAC* strain^23^ that has mural cells labelled in red to generate *Cdh5(PAC)-cre*^*ERT2*^*;Eng*^*f/f*^*;NG2DsRedBAC* mice. The experimental cohort consisted of 166 mice that were of either gender and used at 8–12 weeks of age. The mice were divided into control and *Eng-iKO*^*e*^ groups and were treated either with a vehicle alone or with selected chemical inhibitors as specified in the source data table.

Mice received tamoxifen (2.5 mg day^−1^, total volume 50 μl per injection) by intraperitoneal injection for 5 consecutive days to deplete endoglin in endothelial cells. Tamoxifen was purchased from Sigma-Aldrich (T5648) and dissolved in 1:4 ethanol (Merck, 107017) and corn oil (Sigma-Aldrich, C8267) vehicle. Animals were subsequently collected on day 9.

Intraperitoneal injection of either the vehicle (DMSO or PBS), C381 (10 mg kg^−1^ of animal body weight, volume 50 μl) or VAD044 (5 mg kg^−1^ of animal body weight, volume 50 μl) was administrated to Eng-*iKO*^*e*^ mice for 2 consecutive days. Injections started on day 2 after gene deletion. Control animals received vehicle alone. Animals were subsequently collected on day 9.

The contrast agent used for fULM experiments (Sonovue Bracco, reconstructed in 0.3 ml saline) was injected intravenously in the tail of the mouse by a push syringe (KD Scientific) leading to a continuous injection rate of 800 μl h^−1^. The highest injection volume allowed by international recommendations for a mouse of 25 g is 0.62 ml^49,50^. Here, the injection of <0.4 ml corresponded to a maximum 22% change in a total blood volume of 1.8 ml. The MB solution was manually mixed in the syringe during the resting time interval by waving a magnet inside to ensure the temporal stability of the number of MBs that were injected. Facial left whiskers of mice were brushed manually at a 2-Hz frequency to create local hyperaemia in the somatosensory cortex. The whisker stimulation protocol included 30 s of rest, 30 s of stimulation and 10 s of rest. This pattern was repeated 25 times for fULM acquisitions. Results in Fig. 8b–d were achieved by employing a final pattern with a 10-s duration of stimulation aiming at emphasizing the early response in the power Doppler signal. An fUS acquisition was conducted during 3 stimulation patterns before the start of each fULM experiment to confirm the haemodynamic response to whisker brushing.

For fUS experiments, the whisker stimulation protocol included 50 s of rest and 10 s of stimulation or 30 s of rest and 30 s of stimulation. These patterns were repeated 10 times.

To analyse the retinal vasculature, adult mice were killed, and eyes were removed and prefixed in 4% paraformaldehyde (PFA) in PBS for 10 min at room temperature. We dissected the retinas, post fixed them in 4% PFA in PBS overnight and then processed them for immunohistochemistry.

Mouse brains were collected in physiological salt, fixed overnight in 0.2% paraformaldehyde in 0.1 M phosphate buffer (PB) with 0.12 M CaCl_2_ and 4% sucrose, washed in buffer only and then in PB with 15% sucrose, both overnight at 4 °C. Brains were then incubated in PB with 15% sucrose and 7.5% gelatin for 1 h at 30 °C, immediately frozen in liquid nitrogen-chilled isopentane and stored at −80 °C until sectioned. We used 25-µm sections for staining.

### Immunofluorescence staining

For whole-mount immunofluorescence staining of retinas, eyes were fixed in 4% paraformaldehyde in PBS at r.t. for 10 min, retinas were dissected and post fixed overnight in 4% paraformaldehyde in PBS at 4 °C overnight, and then processed for staining.

Biotinylated *Griffonia simplicifolia* lectin (isolectin-B4) (B-1205, Vector Laboratories, 1:50) combined with streptavidin Cy-5 (PA45001, Sigma-Aldrich, 1:100) was used to stain endothelial cells, and rat anti-mouse endoglin (120401, BioLegends, 1:100) combined with donkey anti-rat Alexa 488 (A-21208, Invitrogen, 1:250) was used to check endoglin deletion. FITC-conjugated anti-α-SMA (clone 1A4) (F3777, Sigma-Aldrich, 1:100) was used to stain SMCs.

Frozen brain sections were fixed in cold acetone for 10 min and then washed with PBS. They were permeabilized with 0.2% Triton X-100 (0694, VWR) for 5 min at r.t. and then blocked for 2 h at r.t. with blocking solution (PBS pH 7.4, Triton X-100 0.1%, chicken serum 5%). Primary antibodies were used in PBS with 2% bovine serum albumin (BSA) (A9418, Sigma) and included rat anti-mouse endoglin (120401, BioLegends, 1:100) to stain endothelial cells and validate endoglin deletion, FITC-conjugated anti-α-SMA (clone 1A4) (F3777, Sigma-Aldrich, 1:100) to stain SMCs, and EPs and goat anti-mouse aminopeptidase N/ANPEP (cd13) (R&D systems, AF2335, 1:100) to stain pericytes. After washing with PBS, sections were incubated for 2 h at r.t. with secondary antibodies diluted in blocking buffer. The secondary antibodies used were donkey anti-rat Alexa 488 (A-21208, Invitrogen, 1:250) and donkey anti-goat Alexa 555 (A-21432, Invitrogen, 1:250). Endothelial cells were labelled with biotinylated isolectin-B4 (B-1205, Vector Laboratories, 1:50) before mounting the sections with DAKO mounting medium (S3023, Dako).

### Transmission electron microscopy

Isolated retinas were incubated in fixative buffer 1 (0.1 M sodium-cacodylate-buffered solution, pH 7.4, with 2.5% glutaraldehyde and 2% PFA) (16210, 15710, Electron Microscopy Sciences) for 1.5 h at r.t. They were subsequently rinsed twice with 0.1 M sodium-cacodylate-buffered solution and post fixed in fixative buffer 2 (1% osmium tetroxide and 1.5% potassium ferrocyanide in 0.1 M sodium cacodylate) for 1 h on ice. Retinas were washed twice with demineralized water and then rinsed twice with 70% ethanol. Subsequently, retinas were dehydrated overnight in 70% ethanol and incubated sequentially in 80%, 90% (10 min each step) and 100% (2 steps of 15 min and 1 step of 30 min) ethanol.

Tissues were infiltrated with mixtures of Epon LX-112 (21210, Ladd Research Industries) at concentrations of 25%, 50% and then 75% in anhydrous acetone for 30 min. Subsequently, they were infiltrated with pure Epon LX-112 for 2 h, with 1.5 h at r.t. and the last 30 min at 60 °C. Next, the samples were mounted in BEEM flat embedding moulds (AGG3654, Agar Scientific), embedded in Epon LX-112 and polymerized for 48 h at 60 °C.

Ultrathin sections of 100 nm were cut using a diamond knife (Diatome) and collected on single slot copper grids (G2010-Cu, Sciences Services) covered with a formvar film and carbon layer. Sections were post stained with an aqueous solution of 7% uranyl acetate for 20 min, followed by Reynold’s lead citrate for 10 min. Imaging was performed at an acceleration voltage of 120 kV using a Tecnai G2 Spirit BioTWIN TEM (Thermo Fisher) equipped with an FEI 4k Eagle CCD camera, or on an FEI Tecnai G2 12 TWIN electron microscope equipped with a Gatan OneView camera (Gatan). Image mosaics were recorded with binning 2 at ×6,500 or ×6,800 magnification, corresponding to a 3.4-nm and 3.25-nm pixel size at the specimen level. Data were collected and stitched together using automated acquisition and stitching software^51^. Virtual slides were analysed using Aperio ImageScope viewing software v.12.4 (Leica Biosystems).

### Morphometry and quantitative analysis

Complete high-resolution 3D renderings of whole-mount retinas and brain were acquired using a laser scanning microscope (SP5, Leica).

For the retinal vascular network, vessel length, diameter and branch points were manually quantified using ImageJ software (US National Institutes of Health) in 4 fields per retina of 2.4 mm^2^.

For pericyte detachment from the endothelium, we analysed/characterized three types of mural cell in *NG2DsRedBAC* retinas: EPs on proximal branches of the arterioles which covered most of the endothelium, MPs with processes that extensively covered the capillary segments but did not exhibit the regular repeating banding pattern of EPs, and TSPs in the capillary bed that possessed long thin processes. All pericyte subtypes had a clear protruding ovoid soma. We analysed 2D, average-projected confocal images of individual pericytes. ImageJ software was used to measure the roundness of the pericyte cell body. Roundness was calculated as 4 × area/(π × sqr(major axis)). A minimum of 46 pericytes were analysed in at least 3 mice.

Quantification of smooth muscle cell coverage was performed using ImageJ software by dividing the co-localized smooth muscle cell area and the IB4-stained area by the total IB4-stained area.

### Vessel permeability assay

Lysine-fixable cadaverine conjugated to Alexa Fluor-555 (500 mg for 20 g of mouse body weight) (Invitrogen) was injected intravenously into the tail vein of adult mice. Circulation time was 2 h. Animals were collected through perfusion of HEPES saline buffer for 1–2 min, followed by 5 min perfusion with 4% PFA in PBS, pH 7.2. Retinas were dissected, immunostained with biotinylated isolectin-B4 (B-1205, Vector Laboratories, 1:50) and then analysed by confocal microscopy (SP5, Leica).

### Ex vivo retina electrostimulation

Whole retinas were mounted and maintained in an extracellular solution (140 mM NaCl, 25 mM glucose, 5.5 mM KCl, 1.8 mM CaCl_2_, 1 mM MgCl_2_, 10 mM HEPES, pH 7.3). Pericytes were stimulated by pressing the electrode filled with the same solution against the cell and by applying voltage pulses (0.02-ms pulses, 10 Hz for 5 s). Resistance of the pipette was measured to define the input voltage (0.02-ms pulses, 10 Hz for 5 s) to be applied to deliver a current intensity increasing from 1 to 3.5 μA. A total of 600 images were recorded in 2.5 min.

### Functional ultrasound (fUS) acquisition

#### Acquisition

Functional ultrasound imaging acquisitions were performed with a 1D linear transducer (15 MHz central frequency, 128 elements, 110 spatial pitch) connected to a prototype functional ultrasound scanner (Iconeus One, Iconeus and Inserm ART Biomedical Ultrasound). Data were acquired by emitting a group of 11 plane waves tilted from −10° to +10°, fired at a 5.5-kHz pulse repetition frequency. The backscattered echoes of each group were summed to get compound images at 500 Hz frame rate. Power Doppler images were computed from blocks of 200 compound images averaged after filtering with the singular value decomposition (SVD) spatiotemporal clutter filter^52^, removing the first 60 singular values to discard tissue motion. Each pixel of the final power Doppler image was reconstructed on a 100 × 100 μm^2^ pixel on the plane, and the slice thickness was ~300 μm.

#### Analysis of the relative blood volume in activated areas

Analysis of the relative blood volume in activated areas. Initial activation maps were computed for each animal with a single-subject generalized linear model. The stimulus pattern presented above was convolved by a canonical haemodynamic response function (HRF), and statistical parametric maps (SPM) were generated for each scan. Data pre-processing consisted of simple linear detrending. The cerebral blood volume temporal response was then estimated from a region of interest (ROI) (1.4 mm diameter) centred on the peak *z*-score of the activation map, which was always located within the somatosensory barrel cortex S1BF area. The cerebral blood volume (CBV) curves were then normalized to the baseline. Mean relative CBV values were estimated from the ΔCBV curves at early onset (average between 36 and 44 s) and later during the stimulus (average between 44 and 60 s). The rise time of the responses was estimated from the duration taken to increase the ΔCBV from 20 to 90% of the peak ΔCBV values.

Data processing and analyses were performed using Matlab (MATLAB release 2018a, MathWorks).

### Functional ultrasound localization microscopy

#### Acquisition protocol and image processing

The acquisition protocol was the same as the one used for functional ultrasound. The ultrasound localization microscopy pipeline applied is described in Supplementary Fig. 10. The previous blocks of 200 frames acquired at 500 Hz were first processed to discriminate the tissue from the injected contrast agent by discarding the first 20 eigenvalues of SVD decomposition. Each frame was then spatially interpolated 6 times using a Lanczos interpolation kernel. The final spatial resolution chosen for rasterization was ‘probe spatial pitch/16 × *λ*/16’, corresponding to ‘6.875 μm × 6.5 μm’. MBs were identified as the most intense local maxima of the correlation with a typical point spread function (Gaussian spot). Only MBs resulting in a correlation value >0.7 were kept for the next step of processing.

A spatial second-order polynomial fit was then used to identify the subpixel maxima localization corresponding to the centre of an individual MB. Absolute coordinates obtained were projected onto the grid chosen to be 16 times smaller than the initial resolution.

A classical particle tracking algorithm (https://github.com/tivenez/simpletracker) was employed to track the maxima positions at each time step. Various additional constraints were used: the maximum particle speed was set to 100 mm s^−1^ and MBs needed to be detected on at least 5 successive tracks to avoid rejection. The successive positions of an individual MB were compared to the previous one to derive its velocity vector field.

After estimating each individual microbubble trajectory using the tracking algorithm, a spatial linear interpolation was performed along the track to fill in the pixels crossed by the bubble trajectory between two MB positions in successive frames. This ensures that each pixel along the track receives a value of 1, indicating the presence of the microbubble in that pixel at a specific moment during the experiment. For each pixel, the total number of microbubbles detected per second was then calculated by summing these presence values. This results in a quantitative estimate of MB flow, expressed in MBs px^−1^ s^−1^.

#### ULM image construction and data analysis

Dynamic MB count and velocity maps were computed by accumulating successive 2-s time windows of MB trajectories (no overlapping between windows) at each time step. If no bubbles were detected in a pixel during this accumulation time, the speed was set to 0 for this time window. The square root of the density is displayed in Fig. 2a,b to visually adjust the image dynamic range to the smallest vessels.

#### Automatic segmentation of vessels

To automatically delineate vessels, a Frangio vesselness filter (Matlab function fibermetric) is applied to the square root of the MB count map multiplied by the normalized velocity map. First, arterioles and venules from the upper right cortex were discriminated by analysing the sign of the speed vector in the *z* axis. Arteries carry oxygen-rich blood deep into the brain at a positive speed along the *z* axis, while venules flow out of the brain at a negative speed along the *z*axis. Finally, these vessels were extracted using the bwpropfilt function in Matlab, fixing a threshold on the length along the major axis to identify only long-enough vessels.

#### Comparison of the MB velocity and MB flow in arterioles and venules

The averaged MB flow and speed images (Fig. 2a,b) provided information on the haemodynamics of vessels with a 6.5-µm resolution over a brain-wide field of view, allowing multiple haemodynamic and functional quantifications.

For each vessel of the left cortex, manual measures of the speed and the number of MBs detected at various depths were extracted, leveraged on a profile line orthogonal to the vessel. Measures from one representative control and one *Eng-iKO*^*e*^ mouse are shown in Fig. 2. At least 10 vessels were analysed and averaged per mouse in Fig. 7f. The dispersion of speed values observed in each animal (Supplementary Fig. 8d) was linked to the distribution of sizes of the brain cortex’s vessels. Resulting mean flows and mean speeds were compared at various depths in the cortex, with separation between venules and arteries.

#### fULM data construction and spatial resolution estimation

Dynamic ULM images were built by accumulating 2 s of temporal frames, that is, 5 blocks of 200 ultrasound compounded frames. This set of MB flow and velocity images corresponded to the ‘raw temporal ULM data’ that could be described in the form of a matrix (*Nx*, *Nz*, *Nt*) with *Nx* = 2,150 (spatial extention of 14 mm along the probe’s transducer line with a 6.5-µm final resolution), *Nz* = 1,386 (spatial extension of 9 mm in depth) and *Nt* = 875 (30 min of acquisition with a 0.5-Hz acquisition rate, with *Nt* = pattern duration (70 s) × number of patterns (25)).

From these raw ULM data, a ‘pattern-averaged temporal ULM data’ matrix of size (*Nx*, *Nz*, *Nt*’) with *Nt*’ = pattern duration (70 s, 35 ULM images) built by accumulating the density and speed of the 25 patterns corresponding to the same time point within the temporal pattern (Supplementary Fig. 10)^27^. A mean MB flow and velocity dynamic mapping over the averaged stimulation pattern was built this way with one map every 2 s during the 70-s recombined stimulation pattern.

#### Construction of activation maps in fULM

The acquisition dataset was split into two subsets (Supplementary Fig. 8), one corresponding to baseline periods (0–30 s within each repeated stimulation pattern) and another corresponding to stimulation periods (30–60 s within each repeated stimulation pattern). MB flow and velocity maps were computed for these two subsets. Maps shown in Figs. 2a,b and 5 and Supplementary Fig. 8 were the resulting baseline MB flow and velocity images.

Activation maps showing the local increase in MBs during the whisker stimulation were computed as the difference of the baseline and activation density maps normalized by the baseline density (Supplementary Fig. 10c).

Furthermore, we displayed all raw trajectories of MBs corresponding to accumulated 4-s time windows before stimulation and during the activation pattern (Fig. 5b). These so-called ‘MB tracks’ were superimposed on the cumulative activation map corresponding to the relative MB flow increase averaged from the start of the stimulation (*t* = 30 s) to the actual time window. This ‘raw track data’ visualization allowed for more insight into how the activation starts in the barrel cortex, with a clear initial appearance of new MB tracks in vessels surrounding the activated arteriole (30–34 s) and a progressive increase in MB tracks propagating to the upper layers of the cortex in the upcoming time windows (Fig. 5b, 34–38 s, 38–42 s, 42–46 s, 54–58 s).

#### Temporal analysis pipeline

The temporal analysis pipeline is described in Supplementary Fig. 10.

ULM MB flow and velocity maps corresponding to each time step within the stimulation patterns were first built at 0.5 Hz. To avoid any potential bias due to global variations in MB injections, only the stimulation patterns occurring during stable global MB flow injection were chosen manually and used for the analysis. If the global variation in MB flow in the whole brain was more important than the variation due to the whisker stimulation, the current pattern was not taken into account for the reconstruction of the global averaged signal. Among the whole set of 25 stimulation patterns, a typical number of *N* = 20.9 ± 2.7 stimulation patterns was kept for the analysis for all animals.

In agreement with previous results obtained using optical imaging^53^, the relative increase in MB count was higher in the small vessels surrounding the arteriole than in the arteriole itself (Supplementary Fig. 10c). To compare fULM responses to the whisker stimuli, an area with constant size was selected manually in the stimulated area of the right cortex corresponding to the somatosensorial S1BF area for each animal. In this ROI, an automatic extraction of venules and arterioles was performed with a vessel filter (vesselness 2D) to keep only penetrating vessels (Supplementary Fig. 10d and Supplementary Video 1). Arterioles were then split into two distinct parts: an upper part ranging from 0 μm (taken as the distance from pial vessels) to 400 μm depth, and a lower part ranging from 400 μm depth to the lowest part of the vessel.

The MB flow map was averaged spatially at each time step over all the points composing the ROI. The temporal MB flow profile for this ROI was normalized by the baseline and was called ‘the activation signal’.

This signal was summed over the two groups (control and *Eng-iKO*^*e*^ mice) to build the activation signals of Fig. 5c,f. The standard deviation of each time point was computed as the mean spatial standard error of all animals.

For both control and *Eng-iKO*^*e*^ activation signals, the MB flow activation measures at early stimulation times (30–40 s) and late response (40–60 s) were compared.

#### Quantification of haemodynamic response recovery

To compare fULM activation signals to the whisker stimuli in the control group (*n* = 11), the *Eng-iKO*^*e*^ (*n* = 12) group and the C381-treated group (*n* = 12) (Fig. 8e), a region of interest was manually selected for each acquisition in the stimulated area of the right cortex corresponding to the somatosensorial S1BF. In each acquisition, some temporal patterns were considered as not sufficiently correlated with the stimulation pattern and were not taken into account in the temporal averaging for MB activation’s signal construction. For this purpose, the correlation of the arterioles’ MB count signal with the stimulation pattern in the selected region of interest was computed and a threshold of 0.3 was chosen. To avoid the presence of non-stimulation-related increase in MB count in the averaged signal, the correlation of a non-stimulated area selected in the contralateral cortex region was manually drawn and its average MB count signal was extracted. Only patterns for which this correlation was between 0.5 and −0.5 were kept. Finally, only acquisitions whose MB count activation signals averaged over the stimulation pattern reached a 10% increase were kept. We built the final activation signal by averaging the MB count signal of animals from each group (*n* = 7 in the control group, *n* = 10 in the *Eng-iKO*^*e*^ group and *n* = 9 in the C381-treated group).

### Backscattering and evaluation of vessel tortuosity

We estimated the amplitude of the signal backscattered by each microbubble after its insonification to build the backscattering ULM images obtained in Fig. 3. The average backscattering coefficient estimated for each pixel provided valuable information about the position of the MBs in the out-of-plane *y*axis^33^. These backscattering maps (Fig. 3a–d) provided an imaging parameter shown to be more suitable than MB flow maps to detect microvessels and to measure vessel diameters (Fig. 3a). Such backscattering ULM images were built for each animal of the two groups.

Arteries longer than 0.5 mm were automatically selected within a square ROI of 4 mm² (8 ± 2 vessels per ROI, mean ± s.d.) located in the cortex, either in the left or right hemisphere. For each selected artery, only tracks longer than 0.1 mm were kept for quantifying tortuosity. The angular variation of the speed vector field within each individual vessel was directly linked to the tortuosity of the vessel. We quantified the standard deviation of the angular distribution of the velocity vector field of some arteries in the upper right cortex of a control and an *Eng-iKO*^*e*^ animal, providing a method for tortuosity assessment in cerebral brain vessels.

### Statistical analysis

We performed statistical analyses with Prism-9 software (GraphPad) using two-tailed, unpaired Student’s *t*-test or one-way analysis of variance (ANOVA) for multiple comparisons. We used Dunnett’s test for post hoc pairwise comparisons. Results are expressed as mean ± s.e.m. *P* values are shown on the graphs. Sample sizes were determined on the basis of a power analysis using a significance level of 0.05 and a power of 0.8 to ensure adequate statistical validity, depending on the effect size of the experiment.

### Reporting summary

Further information on research design is available in the Nature Portfolio Reporting Summary linked to this article.

## Supplementary information

Supplementary Information Supplementary Figs. 1–11.

Reporting Summary

Supplementary Video 1 Automatic segmentation, discrimination and quantification of microbubble velocity in arterioles and venules of the right cortex. After segmentation, microbubble flow and velocities are then automatically measured at various predefined depths.

Supplementary Video 2 Tortuosity detection and microbubble velocity vector quantification in altered vessels of *Eng-iKO*^*e*^ mice. In grey scale, the ULM image of local backscattering amplitude. Superposition in colour scale, independent microbubble trajectories and local speed over time revealing the local tortuosity of a small vessel deep seated in the thalamus.

## Source data

Source Data Figs. 1–8 All data from graph + animal label and number.

## Data Availability

Ultrasound localization microscopy backscattering images and projected velocity images from 35 mice are available from Zenodo at 10.5281/zenodo.15280877 (ref. ^54^). Two 3D (2D + time) ULM datasets are also provided: one from a whiskers stimulation experiment in a control mouse and one in an *Eng-iKO* mouse. Low-level acquisition can only be shared upon request, with the written agreement of INSERM. The confocal and TEM images as well as electrophysiology datasets are available from the corresponding authors on reasonable request with the consent of INSERM or Leiden University Medical Centre. Source data are provided with this paper.

## Code availability

MATLAB codes (R2022b) developed in-house were used to perform ULM analysis including vesselness filtering (available on Mathworks file exchange https://www.mathworks.com/matlabcentral/fileexchange/24409-hessian-based-frangi-vesselness-filter (ref. ^55^)) and the tracking algorithm (simpletracker available on GitHub at https://github.com/tinevez/simpletracker (ref. ^56^)). Custom MATLAB scripts were employed to analyse these data. A MATLAB code to open ULM data, display density and velocity maps as well as to construct functional ULM activation is provided on Zenodo at 10.5281/zenodo.15280877 (ref. ^54^). Processing codes for the raw data used for the collection of ULM data are protected by INSERM and can only be shared upon request, with the written agreement of INSERM.
