# Publisher Correction: Non-invasive characterization of pericyte dysfunction in mouse brain using functional ultrasound localization microscopy

**DOI:** 10.1038/s41551-025-01494-6

**Published:** 2025-08-11

**Authors:** Jérémy H. Thalgott, Nicolas Zucker, Thomas Deffieux, Marit S. Koopman, Alexandre Dizeux, Cristina M. Avramut, Roman I. Koning, Hans-Jurgen Mager, Ton J. Rabelink, Mickaël Tanter, Franck Lebrin

**Affiliations:** 1 Einthoven Laboratory for Vascular and Regenerative Medicine, Department of Internal Medicine (Nephrology), Leiden University Medical Centre, Leiden, the Netherlands; 2 Institute Physics for Medicine Paris, INSERM U1273, ESPCI Paris-PSL, CNRS UMR8063, Paris, France; 3 Department of Cell and Chemical Biology, Electron Microscopy Facility, Leiden University Medical Centre, Leiden, the Netherlands; 4 Department of Pulmonology St Antonius Hospital, Nieuwegein, the Netherlands

**Keywords:** Cerebrovascular disorders, Ultrasonography

Correction to: *Nature Biomedical Engineering* 10.1038/s41551-025-01465-x, published online 30 July 2025.

In the version of the article initially published, the caption for Fig. 5d–h read as follows: “**d**,**e**, Quantification of the MB flow increase in early (**d**) and late (**e**) phases of the stimulation in control (*n* = 5) and *Eng-iKO*^*e*^ (*n* = 8) mice. **f**, Mean time-series of MB flow increase from 400 to 1,200 μm depth. **g**,**h**, Quantification of the MB flow increase in early (**g**) and late (**h**) phases of the stimulation in control (*n* = 5) and *Eng-iKO*^*e*^ (*n* = 8) mice.” It should have been “**d**,**e**, Quantification of the averaged MB flow increase (control *n* = 4, *Eng-iKO*^*e*^ *n* = 6) mice from 0 to 400 µm depth in early (*n* = 5 samples) and late (*n* = 8 samples) phase of the stimulation. **f**, Mean time-series of MB flow increase from 400 to 1,200 μm depth. **g**,**h**, Quantification of the averaged MB flow increase (control *n* = 4, *Eng-iKO*^*e*^ *n* = 6) mice from 400 to 1,200 µm depth in early (*n* = 5 samples) and late (*n* = 8 samples) phase of the stimulation.” This correction has been made to the HTML and PDF versions of the article.

