# Supplementary material for: Non-invasive characterization of pericyte dysfunction in mouse brain using functional ultrasound localization microscopy
Source: Nat Biomed Eng. 2025 Jul 30;10(2):214–30. doi: 10.1038/s41551-025-01465-x (PMC12920088; doi:10.1038/s41551-025-01465-x)
Supplement: Supplementary file 1 — Supplementary Figs. 1–11. [file 41551_2025_1465_MOESM1_ESM.pdf]

nature biomedical engineering

Supplementary information https://doi.org/10.1038/s41551-025-01465-x

# Non-invasive characterization of pericyte dysfunction in mouse brain using functional ultrasound localization microscopy

In the format provided by the authors and unedited

**Table-of-contents**

Supplementary fig. 1

Supplementary fig. 2

Supplementary fig. 3

Supplementary fig. 4

Supplementary fig. 5

Supplementary fig. 6

Supplementary fig. 7

Supplementary fig. 8

Supplementary fig. 9

Supplementary fig. 10

Supplementary fig. 11

Supplementary Video 1

Supplementary Video 2

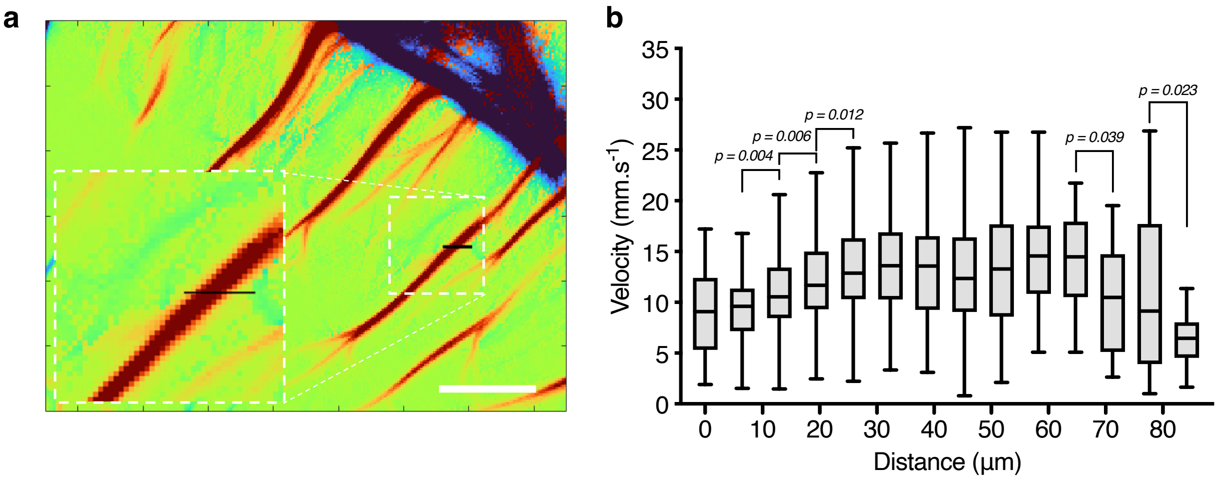


**Supplementary fig. 1 | Estimation of ULM spatial resolution. (a)** Example of a cortical map of the signed MB velocity in arterioles and venules. A higher magnification image in the box shows the selected transverse profile (black) in one representative arteriole (scale bar: 300 µm). **(b)** MB velocity estimates within each pixel as a function of distance along the vessel profile. *P values* result from unpaired two-tailed Student's *t*-tests between each successive box corresponding to each successive pixel. Pixel size: 6.5 µm x 6.5 µm. Colour range between -12 $mm.s^{-1}$ and +12 $mm.s^{-1}$.

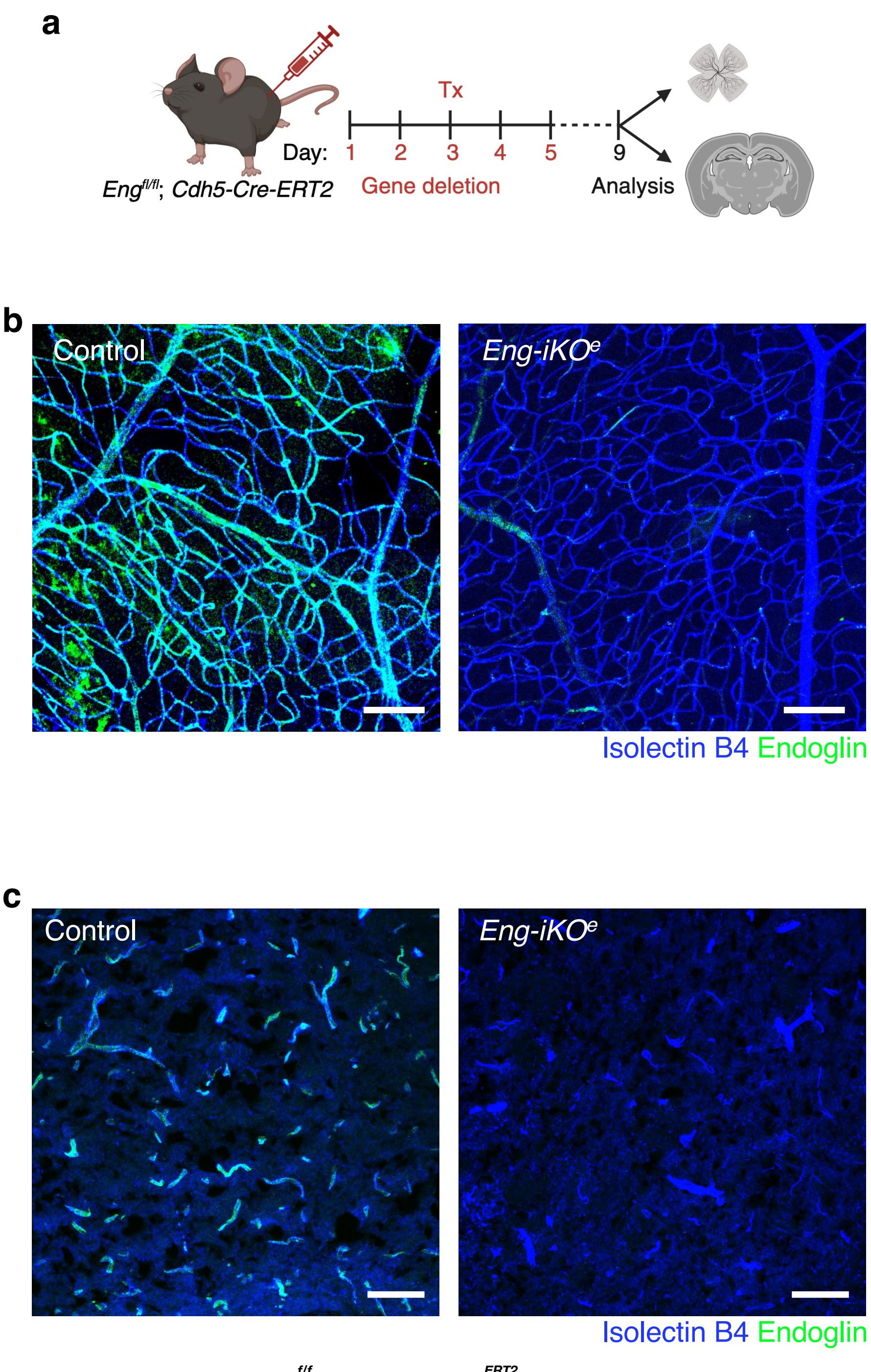


**Supplementary fig. 2 | Endoglin deletion in *Eng*$^{f/f}$; *Cdh5(PAC)-CRE*$^{ERT2}$ at day 9. (a)** Diagram of the tamoxifen injection scheme. **(b)** Confocal images of retinas from control and *Eng-iKO*$^{e}$ mice stained with isolectin B4 (blue) and endoglin (green) (scale bar: 100 μm). **(c)** Confocal images of brain sections control and *Eng-iKO*$^{e}$ mice stained with endoglin (green) (scale bar: 50 μm).

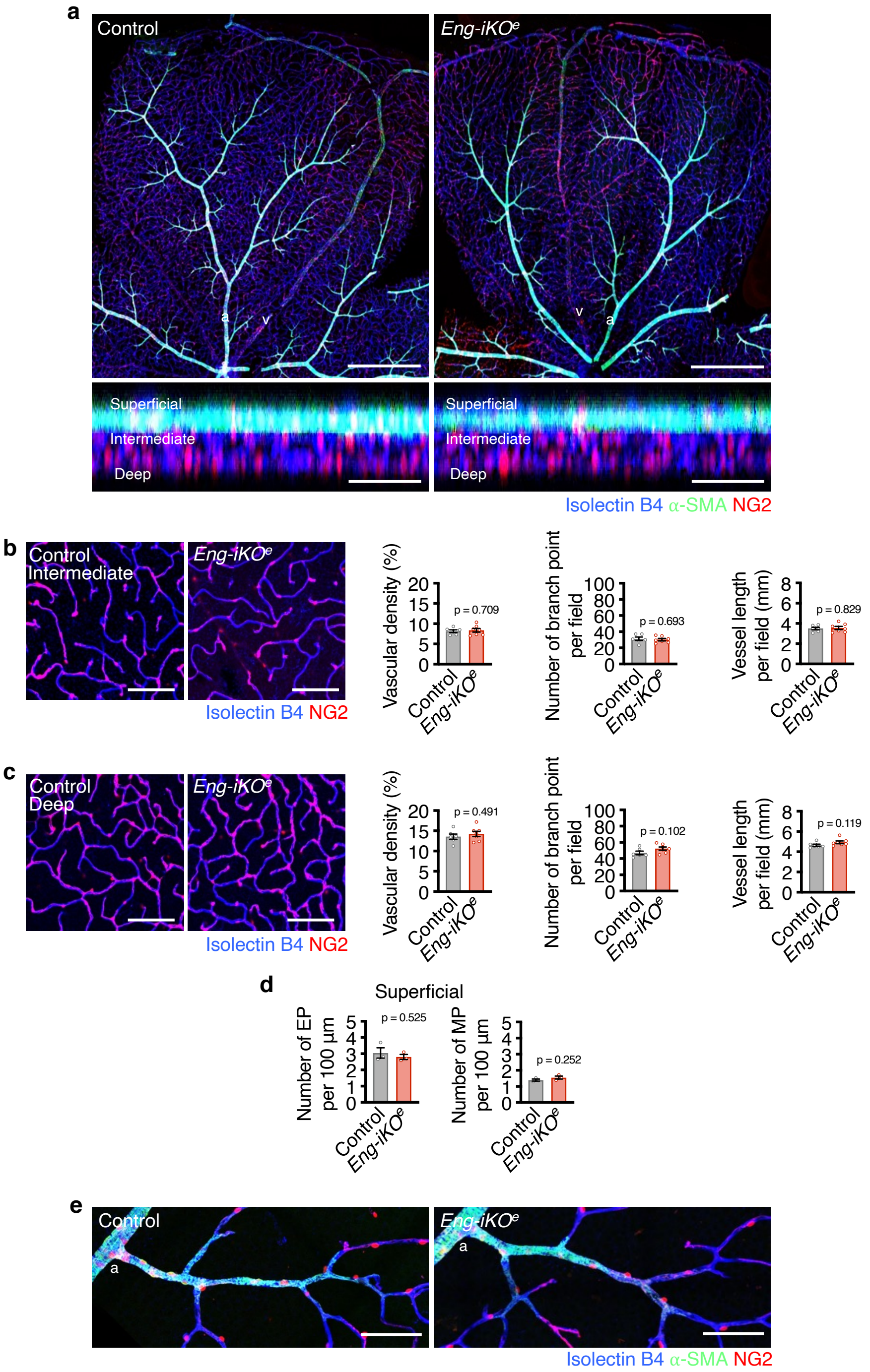


**Supplementary fig. 3 | Retinal vascular layers and mural cell organisation. (a)** Top, confocal images of control and *Eng-iKO*$^e$ crossed with *NG2DsRedBAC* to label mural cells in red stained with α-SMA (vascular smooth muscle cells, green) and isolectin B4 (endothelial cell, blue) (scale bar: 500 µm). Bottom, z-axis projection showing the three different layers (scale bar: 100 µm). **(b)** Left, high magnification images of the intermediate vascular layer and at right, quantification of the vascular density, vessel length and number of branch points in control (n = 6) and in *Eng-iKO*$^e$ (n = 7) mice. **(c)** Left, high magnification images of the deep vascular layer (scale bar: 50 µm) and at right, quantification of the vascular density, vessel length and number of branch points in control (n = 6) and in *Eng-iKO*$^e$ (n = 7) mice. **(d)** Number of pericytes per vessel length in the superficial layer of the retinal vasculature in control (n = 3) and in *Eng-iKO*$^e$ (n = 3) mice. **(e)** High magnification of transitional zone in control and in *Eng-iKO*$^e$ mice showing how α-SMA decreases in intensity from the SMC population to the mesh pericyte population at the ACT zone (scale bar: 100 µm). "a" indicates artery, "v" indicates vein. Error bars show SEM. *P* values result from unpaired two-tailed Student's *t*-tests.

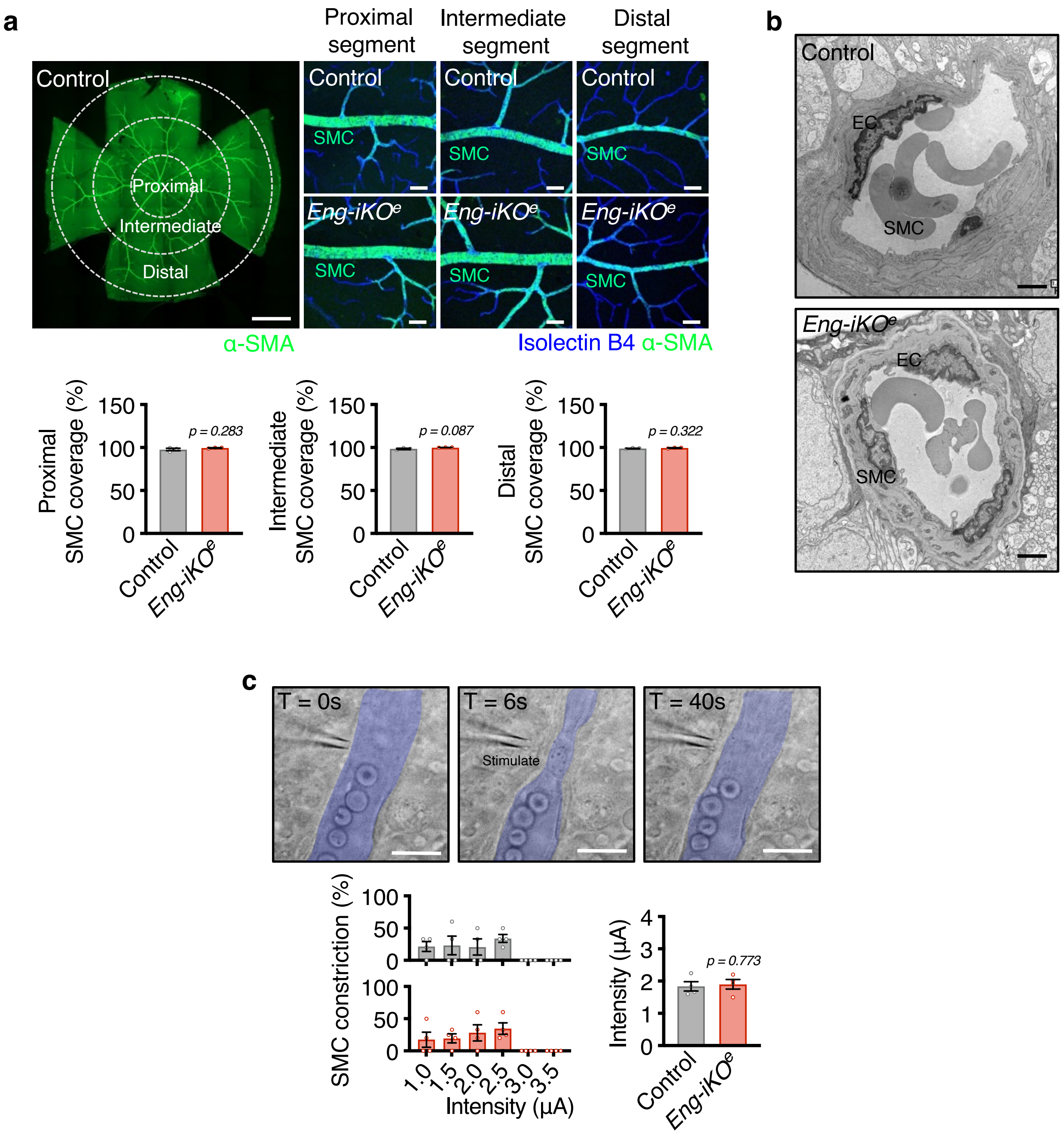


**Supplementary fig. 4 | Smooth muscle cell morphology and function at day 4 after *Eng* gene deletion. (a)** Left, confocal image of a whole retina stained for α-SMA to mark SMCs and contractile pericytes with overlaid scale bar used to divide it into three zones for quantification (scale bar: 500 µm). Right, high magnification images of segments located at the proximal, intermediate and distal zone of the main retinal artery from control and *Eng-iKO*$^{e}$ mice (scale bar: 50 µm). Retinal blood vessels are stained with isolectin B4 (marking endothelial cells in blue) and with α-SMA (labelling SMCs and contractile pericytes in green). Quantification of SMC coverage in the three zones is indicated in control (3, total vessels = 9) and *Eng-iKO*$^{e}$ mice (3, total vessels = 9). **(b)** TEM image showing a retinal blood vessel covered by SMCs in *Eng-iKO*$^{e}$ mice (scale bar: 2 µm). **(c)** At the top, an arteriole with SMCs before, during and after electrical stimulation (scale bar: 20 µm). In the bottom left, the proportion of SMCs inducing a vascular constriction at the indicated current (µA) and in the bottom right, the mean intensity inducing SMC-mediated vessel constriction in control (n = 5, total vessels = 18) and *Eng-iKO*$^{e}$ mice (n = 9, total vessels = 25). Error bars show SEM. *P* values result from unpaired two-tailed Student's *t*-tests.

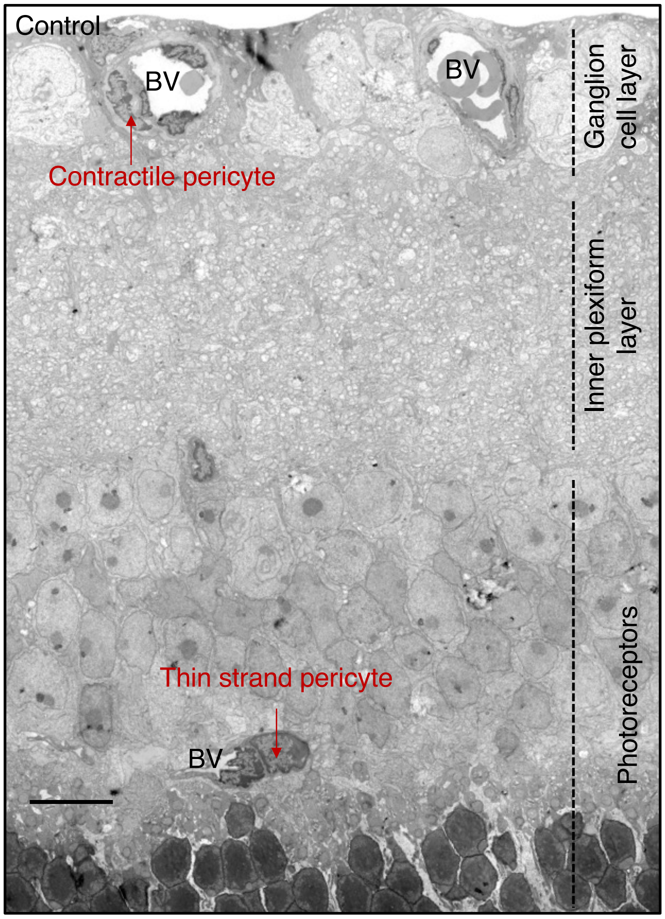


**Supplementary fig. 5 | TEM transversal image** showing contractile EP/MP at the superficial layer and TSP localized in the deep layer of the mouse retina (scale bar: 10 µm).

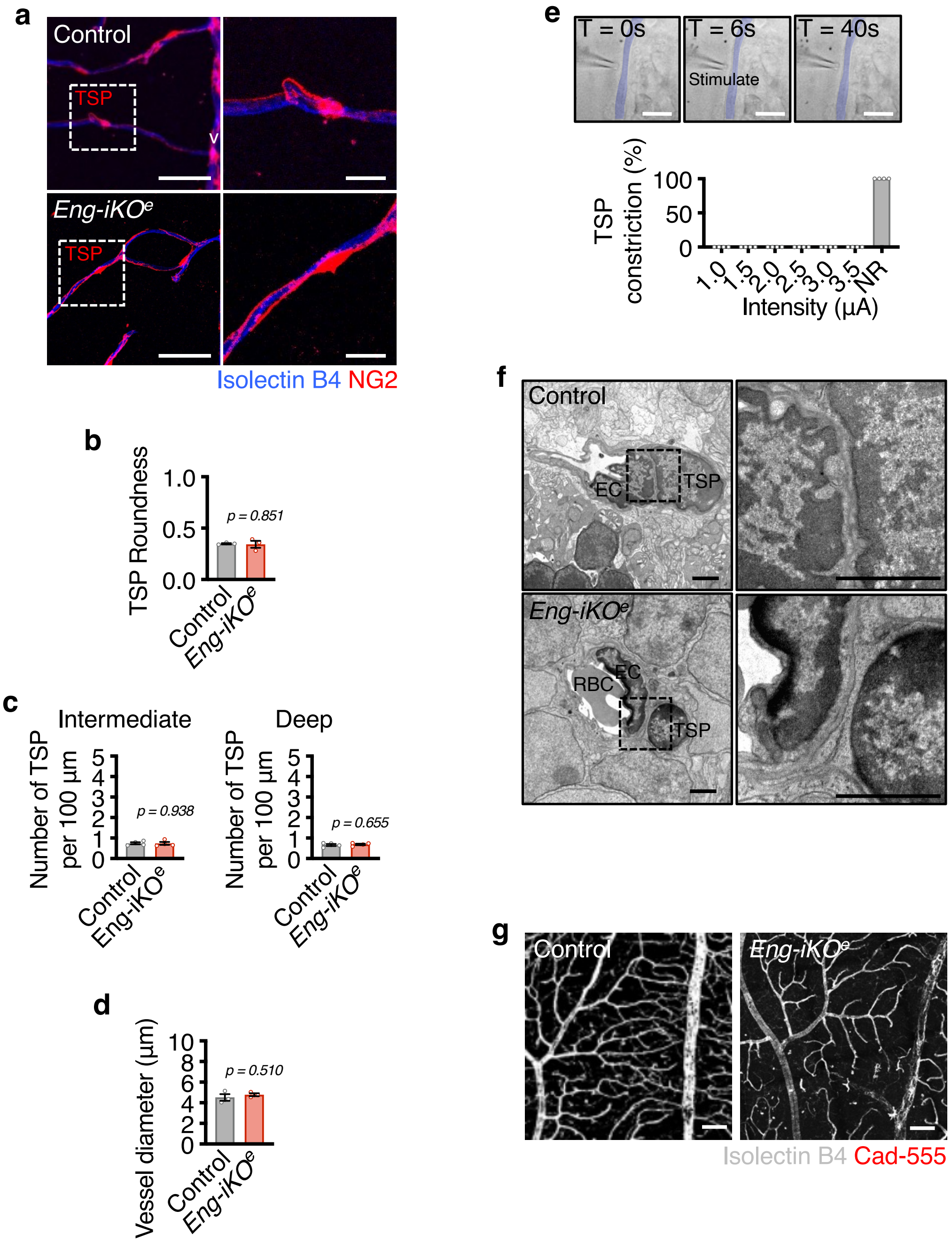


**Supplementary fig. 6 | Thin-Strand Pericytes morphology and function. (a)** Left, confocal images of TSPs labelled in red in mouse retinas of control and *Eng-iKO*$^e$ mice stained with isolectin B4 (endothelial cell, blue) (scale bar: 50 µm). High magnification images in boxes are shown at right (scale bar: 10 µm). "v" indicates vein. **(b)** Quantification of the pericyte roundness in control (n = 3, total TSPs = 80) and in *Eng-iKO*$^e$ (n = 3, total TSPs = 38) mice. **(c)** Number of pericytes per vessel length in the intermediate layer in control (n = 4) and in *Eng-iKO*$^e$ (n = 4) mice and in the deep layer in control (n = 4) and in *Eng-iKO*$^e$ (n = 4) mice. **(d)** Quantification of the vessel diameters in control (n = 3, total vessel = 10) and in *Eng-iKO*$^e$ (n = 3, total vessel = 11) mice. **(e)** At the top, brightfield microscope images of capillaries before, during and after electrical stimulation of a TSP (scale bar: 20 µm). At the bottom, proportion of TSPs inducing a vascular constriction at the indicated current (µA). "NR" indicates no response. **(f)** At the left, representative TEM images of one control and *Eng-iKO*$^e$ TSP (scale bar: 1 µm). At the left, high magnification images in boxes are shown to visualize the tight association though direct contacts between the endothelial cell and the pericyte (scale bar: 1 µm). **(g)** Confocal images of retinas stained with isolectin B4 (endothelial cell, white) from control and *Eng-iKO*$^e$ mice perfused with Cadaverine-Alexa555 (scale bar: 100 µm). Error bars show SEM. *P* values result from unpaired two-tailed Student's *t*-tests.

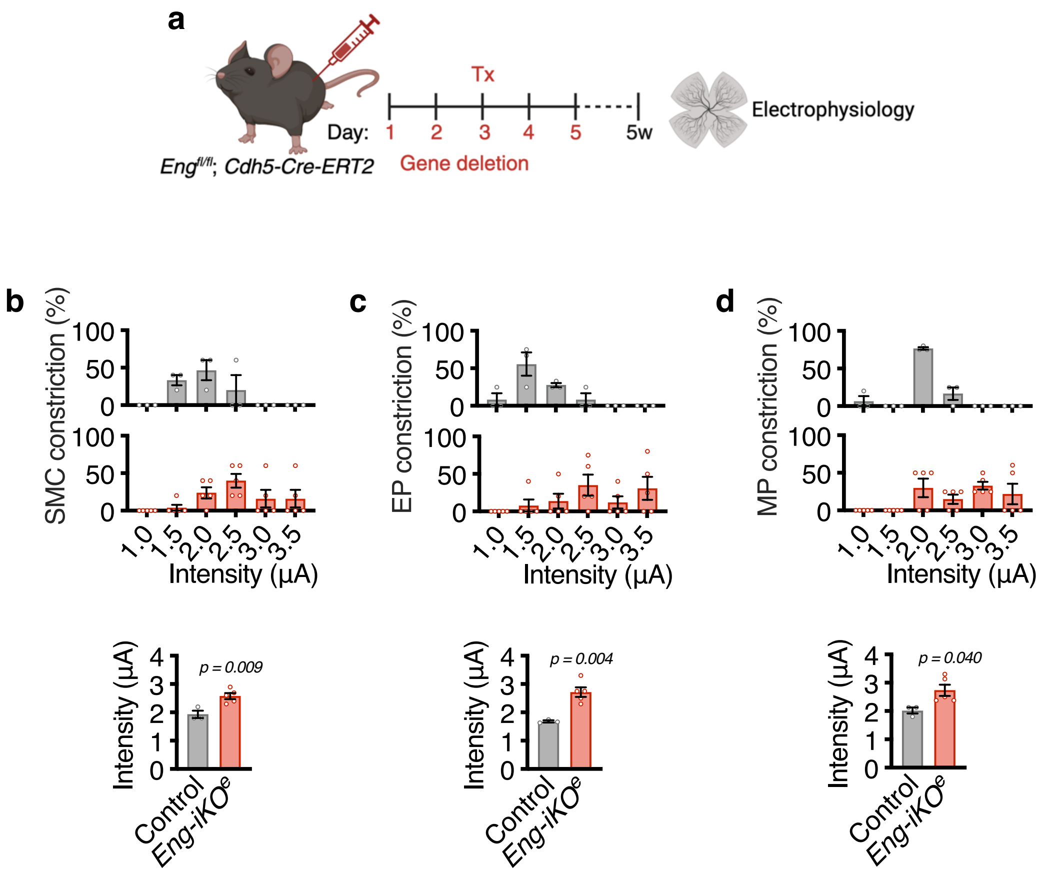


**Supplementary fig. 7 | Mural cells ability to induce a vascular constriction 5 weeks after *Eng* gene deletion. (a)** Diagram of the tamoxifen injection scheme. **(b)** Proportion of SMCs inducing a vascular constriction at the indicated current (µA) and mean intensity inducing vessel constriction in control (n = 3, total SMCs = 15) and *Eng-iKO*$^{e}$ (n = 5, total SMCs = 25) retinas. **(c)** Proportion of EPs inducing a vascular constriction at the indicated current (µA) and mean intensity inducing EP-mediated vessel constriction in control (n = 3, total EPs = 11) and *Eng-iKO*$^{e}$ (n = 5, total EPs = 21) retinas. **(d)** Proportion of MPs inducing a vascular constriction at the indicated current (µA) and mean intensity inducing MP-mediated vessel constriction in control (n = 3, total MPs = 13) and *Eng-iKO*$^{e}$ (n = 5, total MPs = 19) retinas. Error bars show SEM. *P* values result from unpaired two-tailed Student's *t*-tests.

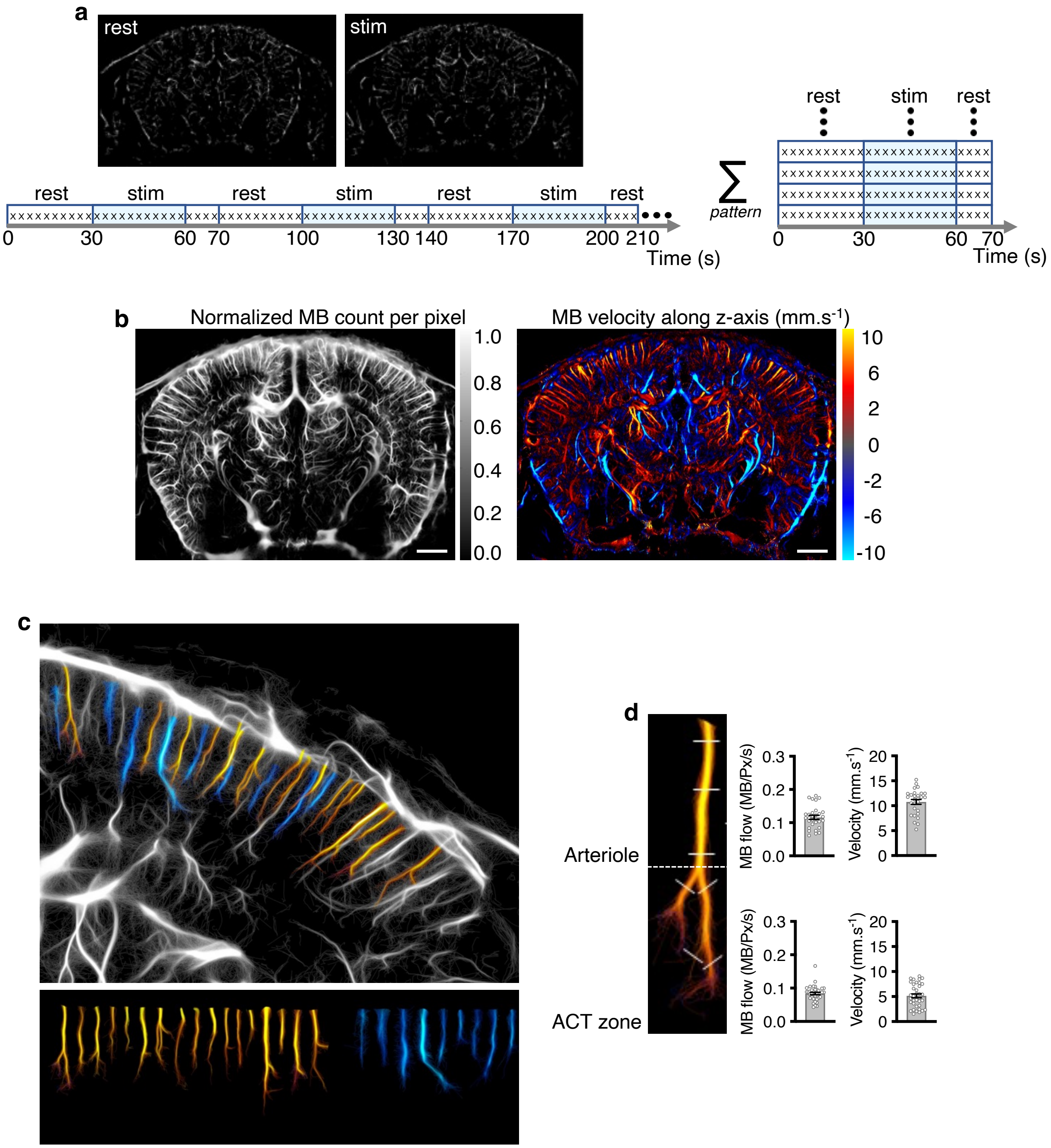


**Supplementary fig. 8 | Construction of baseline density and velocity maps and haemodynamic quantification of segmented vessels. (a)** Schematic of the pattern summation process for the construction of accumulated density and velocity maps. **(b)** Left, ULM image of MB flow (MB/Px/s). Right, ULM image of MB velocity along the z-axis (scale bar: 1 mm). **(c)** Scheme of segmentation of arteries (yellow) and venules (blue) alongside with an example of measurement points (white segments). **(d)** Quantification of MB flow and MB velocity in arterioles (top) and its associated ACT zone (bottom) of a representative animal. Error bars show SEM.

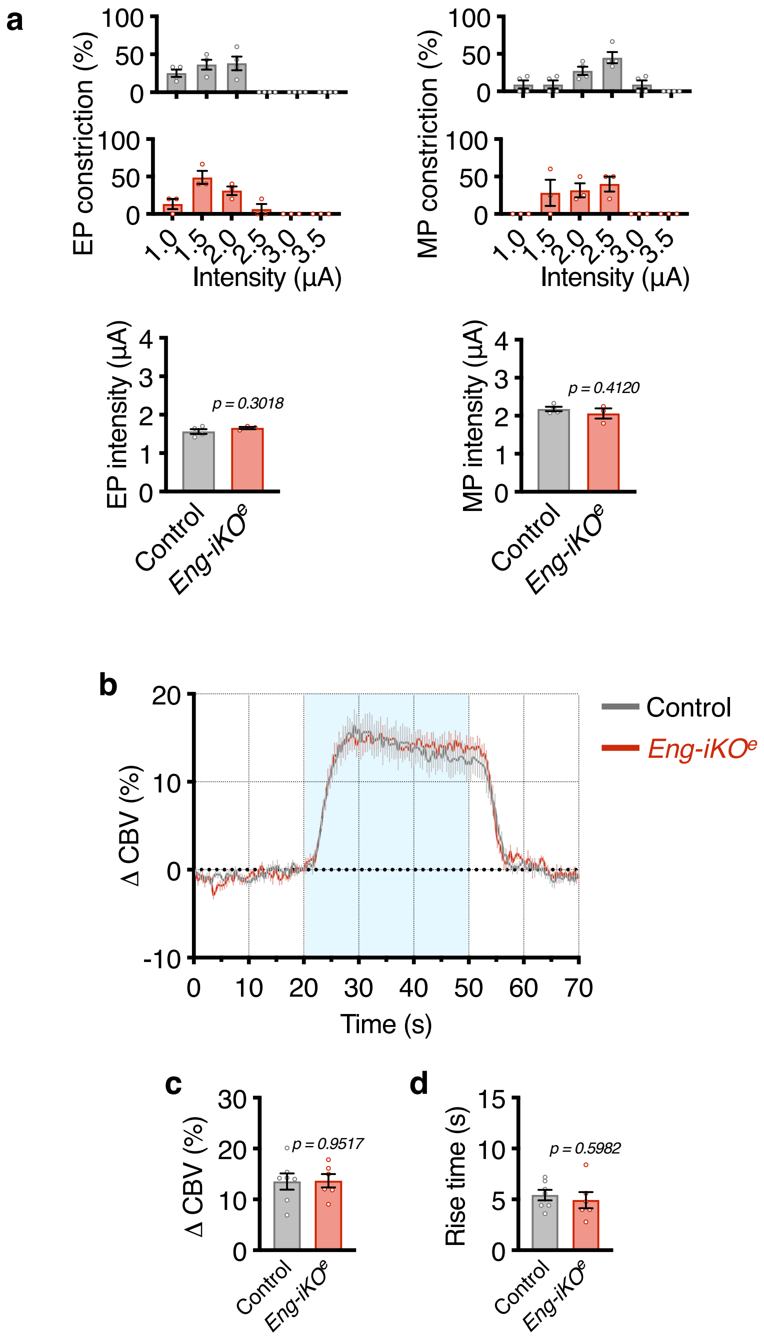


**Supplementary fig. 9 | Pericyte response to electrical stimulation and fUS in whisker-evoked cortical activity in *Eng-iKO*$^{e}$ mice, one day post-gene deletion. (a)** Top left, the proportion of EPs inducing vascular constriction at the indicated current (µA). Bottom left, the mean current intensity required to induce EP-mediated vessel constriction in control (n = 4, total EPs = 30) and *Eng-iKO*$^{e}$ (n = 3, total EPs = 16) mice. Top right, the proportion of MPs inducing vascular constriction at the indicated current (µA). Bottom right: the mean current intensity required to induce MP-mediated vessel constriction in control (n = 4, total MPs = 27) and *Eng-iKO*$^{e}$ (n = 3, total MPs = 13) mice. **(b)** Average haemodynamic response within a chosen region of interest. **(c)** Quantification of the increased response amplitude and **(d)** rise time in both control (n = 7) and *Eng-iKO*$^{e}$ (n = 6) mice. All error bars represent SEM. *P* values result from unpaired two-tailed Student's *t*-tests.

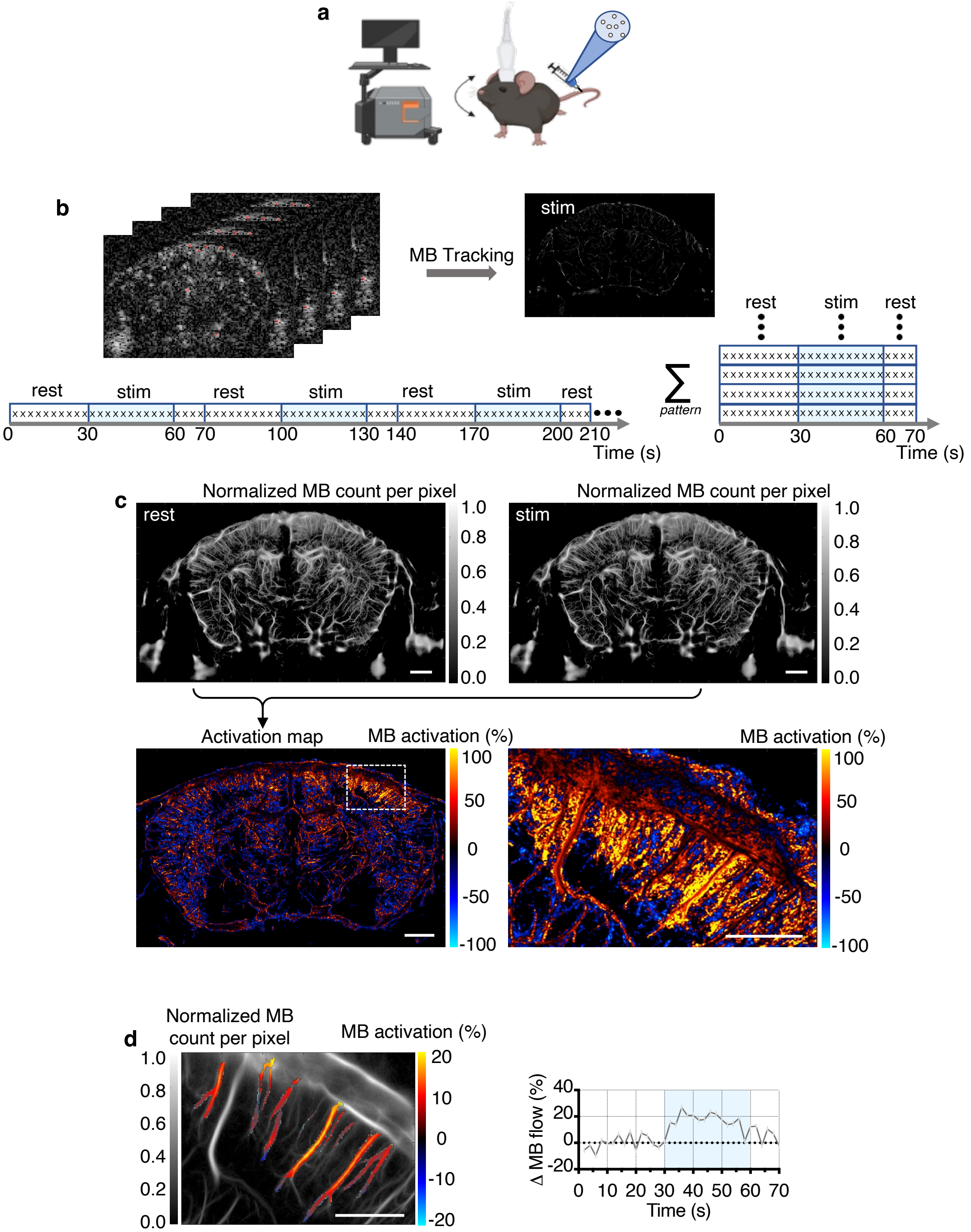


**Supplementary fig. 10 | Transcranial fULM signal analyse pipeline. (a)** Schematic of the experimental setup. **(b)** Diagram of ULM bubble tracking and averaged MB flow map construction for haemodynamic signal construction. **(c)** Up, comparison of baseline MB flow averaged over rest and stim period. Bottom left, construction of an activation MB flow map by calculating the difference between the two previous maps divided by the baseline one. Bottom right, high magnification into the activated somatosensory cortex (scale bar: 1 mm and 500 µm). **(d)** Extraction of stimulated arterioles and creation of a temporal MB flow profile in this area by averaging at each time step the spatial density in the region.

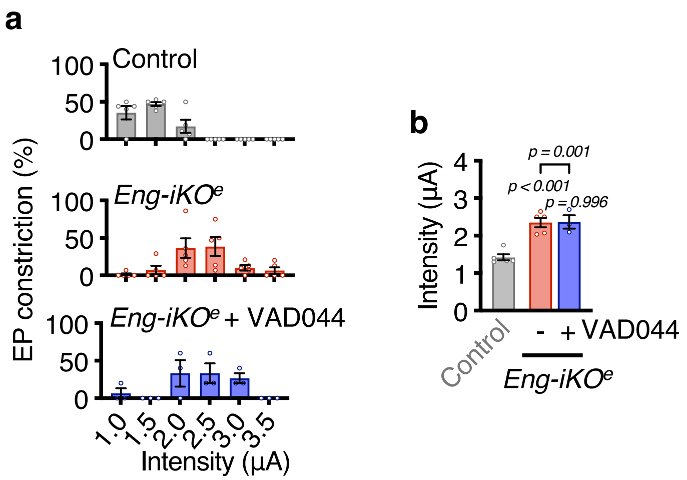


**Supplementary fig. 11 | VAD044 unable to rescue the ability of the pericyte to induce vascular constrictions. (a)** Proportion of EPs inducing a vascular constriction at the indicated current (µA) and **(b)** mean intensity inducing EP-mediated vessel constriction in in control (n = 5, total EPs = 45), *Eng-iKO*$^e$ (n = 5, total EPs = 54) and *Eng-iKO*$^e$ mice treated with VAD044 (n = 3, total EPs = 15). All error bars represent SEM. *P* values result from 1-way ANOVA and Dunnett's post hoc tests comparing the mean of each group to the control group.

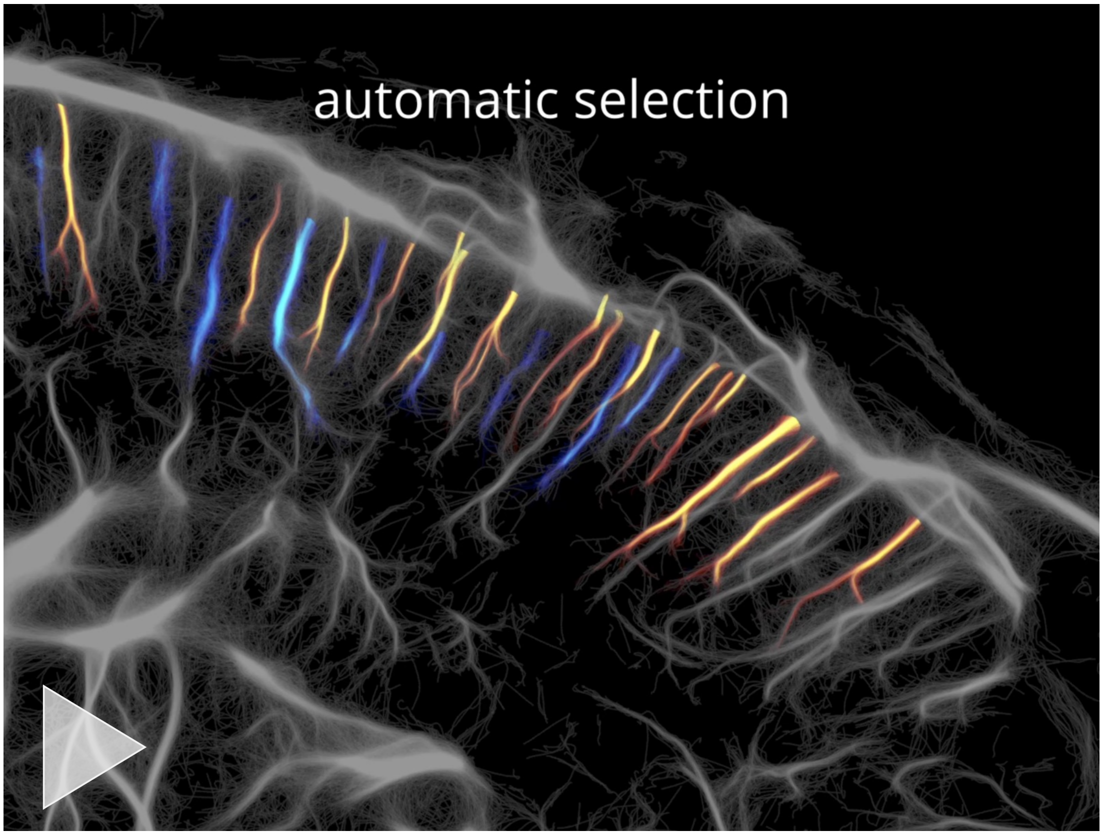


**Supplementary Video 1 | Automatic segmentation, discrimination and quantification of microbubble velocity in arterioles and venules of the right cortex.** After segmentation, micro-bubbles flow and velocities are then automatically measured at various predefined depths.

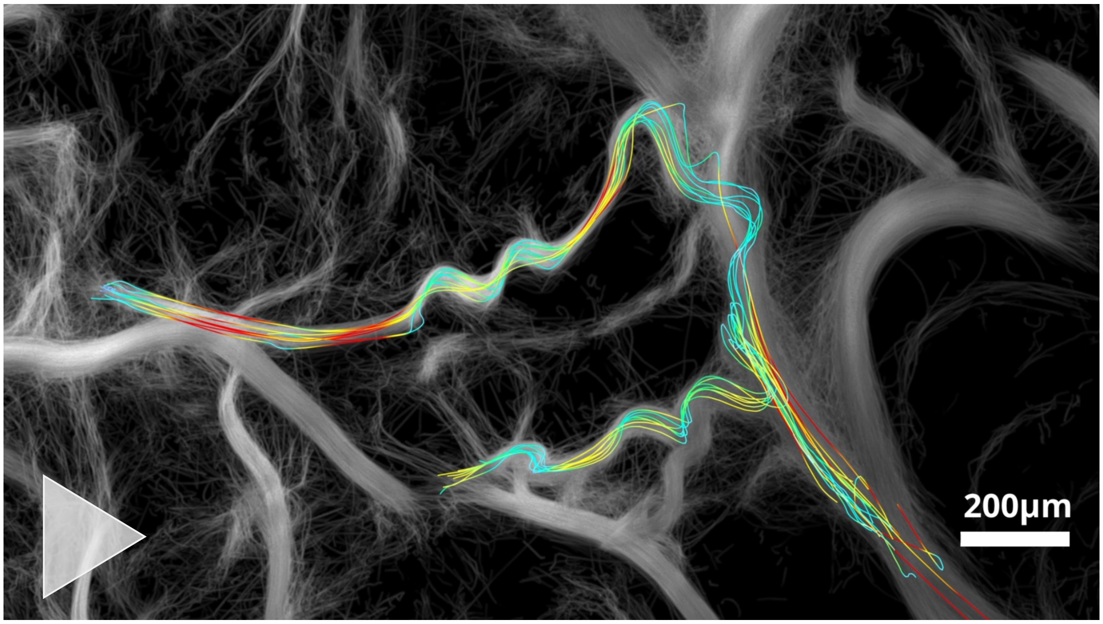


**Supplementary Video 2 | Tortuosity detection and microbubbles velocity vector quantification in altered vessels of *Eng-iKO$^e$* mice.** In grey scale, the ULM image of local Backscattering amplitude. Superposition in colour scale of independent micro-bubbles trajectories and local speed over time revealing the local tortuosity of a small vessel deep seated in the thalamus.
